# Long-term mortality and risk of chronic kidney disease in children following severe malaria complicated by acute kidney injury: a prospective observational study

**DOI:** 10.1016/j.langlo.2026.103984

**Published:** 2026-08-10

**Authors:** Kagan A Mellencamp, Ruth Namazzi, Anthony Batte, Caroline Kazinga, Claire D Liepmann, Michael J Goings, Robert O Opoka, Dibyadyuti Datta, Jie Ren, Robert Kalyesubula, Paul Bangirana, Stuart L Goldstein, Chandy C John, Andrea L Conroy

**Affiliations:** Ryan White Center for Pediatric Infectious Diseases and Global Health (K A Mellencamp PhD, C D Liepmann MD, M J Goings MPH, D Datta PhD, Prof C C John MD, A L Conroy PhD) and Department of Biostatistics and Health Data Sciences (J Ren PhD), Indiana University School of Medicine, Indianapolis, IN, USA; Department of Paediatrics and Child Health (R Namazzi MMed), Child Health and Development Centre (A Batte MMed), Department of Physiology (R Kalyesubula PhD), and Department of Psychiatry (P Bangirana PhD), Makerere University College of Health Sciences, Kampala, Uganda; Global Health Uganda, Kampala, Uganda (R Namazzi, A Batte, C Kazinga MSc, R O Opoka PhD, P Bangirana, A L Conroy); Aga Khan University East Africa Medical College, Nairobi, Kenya (R O Opoka); Center for Acute Care Nephrology, Cincinnati Children’s Hospital, University of Cincinnati College of Medicine, Cincinnati, OH, USA (Prof S L Goldstein MD)

## Abstract

**Background:**

Paediatric acute kidney injury (AKI) is a complication of severe malaria, but its long-term outcomes remain poorly defined in low-income and middle-income countries. We aimed to evaluate the long-term association between paediatric AKI and kidney outcomes and mortality following severe malaria.

**Methods:**

We pooled data from two prospective cohorts of Ugandan children (ie, those aged 6 months–12 years) admitted to hospital with severe malaria between 2008 and 2017. Children with stored admission blood samples available for creatinine measurement were included in the analysis. Surviving participants were enrolled in a follow-up study conducted from 2020 to 2023, when kidney function and survival were assessed, using Cox regression with age as the timescale to model mortality risk. Logistic regression was used to estimate the odds of chronic kidney disease (CKD), defined as two or more consecutive low estimated glomerular filtration rates 90 days or more apart, and long-term major adverse kidney events, defined as CKD or death. Adjusted analyses included the following enrolment characteristics: age, sex, height-for-age *Z* score, study site, study cohort, severe malaria group (ie, cerebral malaria, severe malarial anaemia, respiratory distress, complicated seizures, or prostration), and HIV status.

**Findings:**

At enrolment, the median age was 2·5 years (IQR 1·8–3·5), 622 (57·8%) of 1077 were male and 455 (42·2%) were female, with Kidney Disease: Improving Global Outcomes-defined AKI occurring in 431 (40·0%) children. Over a median of 6·5 years (3·7–8·8), 147 (13·6%) of children died, with nearly half of deaths (71 of 147) occurring after hospital discharge. Adjusted odds of long-term major adverse kidney events were higher among children with AKI (adjusted odds ratio [aOR] 3·14, 95% CI 2·23–4·43), driven by a higher mortality risk (adjusted hazard ratio [aHR] 3·36, 95% CI 2·22–5·10) that remained elevated beyond 2 years after the acute episode (aHR 4·53, 1·80–11·37). AKI survivors had higher odds of chronic kidney disease over follow-up (odds ratio 1·77, 1·07–2·93), although not significant after adjustment (aOR 1·47, 95% CI 0·84–2·56; p=0·18).

**Interpretation:**

In this paediatric population, AKI was associated with excess long-term mortality, suggesting AKI is a sentinel event that marks sustained vulnerability to death and highlights limitations of acute-care models in malaria-endemic settings.

## Introduction

Severe malaria remains a leading cause of childhood morbidity and mortality in Africa, despite gains in malaria prevention and acute case management.^1^ Increasingly, evidence suggests that survival following any severe childhood illness does not mark the end of risk, but the beginning of a prolonged period of vulnerability, with substantial mortality occurring after hospital discharge.^2^ However, the determinants of long-term survival after severe malaria, and clinical features that identify children at sustained risk, remain poorly defined.

Acute kidney injury (AKI) is a common and life-threatening complication of severe malaria in children, affecting up to 60% of those admitted to hospital in malaria-endemic settings.^3^ AKI has consistently been associated with increased risk of in-hospital and early post-discharge mortality, chronic kidney disease (CKD), and cognitive and behavioural impairments 1 to 2 years after the severe malaria illness.^4–6^ However, there are few reports on the long-term outcomes of AKI following severe malaria.

Globally, AKI occurs in nearly one-third of paediatric hospitalisations,^7^ with up to 85% of cases occurring in low-income and middle-income countries (LMICs),^8^ where infections are a dominant driver. In high-income settings, increasing longitudinal evidence shows that paediatric AKI is associated with CKD, recurrent hospitalisation, hypertension, and cardiovascular morbidity beyond the acute phase,^9^ but long-term mortality remains low. Comparable long-term data from LMICs are scarce, particularly for infection-associated AKI, for which limited access to diagnostic testing, nephrology care, and post-discharge follow-up could fundamentally alter long-term outcomes.

Malaria-associated AKI often occurs in settings in which health-care resources are constrained and recurrent infections are common. As such, AKI might function not only as a marker of acute illness severity, but also as a sentinel event that identifies children whose long-term survival trajectories are altered by biological injury and health system limitations.

The objective of this study was to evaluate long-term survival and kidney outcomes following malaria-associated AKI. By following up children for up to 15 years after the index illness, we aimed to determine whether AKI during severe malaria is associated with sustained excess mortality and CKD, and to characterise the relative contribution of each, in a malaria-endemic, low-income setting.

## Methods

### Study design and population

The present study used pooled data from two prospective cohort studies of children with severe malaria. The first study was conducted from 2008 to 2013 and enrolled children aged 18 months to 12 years presenting with cerebral malaria or severe malarial anaemia at the Mulago National Referral and Teaching Hospital in Kampala, Uganda.^10^ Participants were followed up for 24 months, with scheduled visits at 6, 12, and 24 months. The second study was conducted from 2014 to 2017 and enrolled children aged 6 months to 4 years presenting at the Mulago National Referral and Teaching Hospital or Jinja Regional Referral Hospital in Eastern Uganda with cerebral malaria, respiratory distress, complicated seizures, severe malarial anaemia, or prostration.^11^ Participants were followed up for 12 months, with scheduled visits at 1 and 12 months, conducted at the hospital to which children were initially admitted, and a home visit at 6 months. Detailed inclusion and exclusion criteria and clinical management for both studies are included in appendix 5 (p 4). From 2020 to 2023, parents of previous study participants, or the participants themselves if aged 18 years or older, were contacted for participation in a cross-sectional follow-up study assessing long-term neurodevelopmental outcomes of severe malaria with a secondary focus on kidney function. Overall, the follow-up period spanned 15 years, beginning at a child’s initial enrolment in their previous study until enrolment in the follow-up study, loss to follow-up, withdrawal, or death. At enrolment in the previous studies and follow-up study, participants underwent a medical history, physical examination, and blood draw. Sex was recorded as a biological variable at the time of enrolment in the previous study, based on caregiver reports. See appendix 5 (p 2) for detailed information on how mortality was tracked over follow-up.

Written informed consent was obtained from parents or caregiver of participants at enrolment, or from the participants themselves, if aged 18 years or older. Assent was obtained from children aged 7 years and older. The study was approved by Makerere University School of Medicine Research Ethics Committee (cohort 1, 2008–033; cohort 2, 2013–141; and follow up, 2018–091), the Uganda National Council for Science and Technology (cohort 1, HS432; cohort 2, HS1522; and follow up, HS2468), the University of Minnesota (cohort 1, 0802M27022 and cohort 2, 1309M42501), and Indiana University (cohort 2, 1412213778A003 and follow up, 1804188283).

### Assessment of kidney function

AKI and CKD were defined using the Kidney Disease: Improving Global Outcomes (KDIGO) consensus criteria.^12,13^ AKI was defined as a 1·5-fold increase in creatinine over estimated baseline using a height-independent approach assuming a normal glomerular filtration rate (120 mL/min per 1·73 m^2^).^14^ Urine output criteria were not applied because urine output monitoring is not standard of care due to challenges in urine collection and limitations in catheter size availability, thus urine output monitoring was not systematically conducted at the time of the studies. The Chronic Kidney Disease in Children Under 25 equation was used to estimate the glomerular filtration rate (eGFR) at scheduled visits,^15^ with low GFR defined as less than 90 mL/min per 1·73 m^2^ and values truncated at 185. CKD was defined as two or more consecutive post-discharge eGFR readings less than 90 mL/min per 1·73 m^2^ at least 90 days apart.^13^ The cumulative burden of kidney function was assessed using the risk of long-term major adverse kidney events, as defined by the National Institute of Diabetes and Digestive and Kidney Diseases, which included CKD or death over follow-up but excluded kidney replacement therapy as no participants received any such therapy.^16^ A description of kidney replacement therapy availability is provided in appendix 5 (p 1). At final follow-up, kidney dysfunction was defined by KDIGO CKD risk categories, based on serum creatinine eGFR and urine albumin-to-creatinine ratio,^13^ with participants with a moderately increased or greater risk of CKD (ie, eGFR <60 mL/min per 1·73 m^2^ or urine albumin-to-creatinine ratio ≥3 mg/mmol) characterised as having kidney dysfunction.^17^ Because serum creatinine might mask kidney dysfunction due to muscle loss during prolonged illness or malnutrition,^18^ cystatin C-based and creatinine-cystatin C combined Chronic Kidney Disease in Children Under 25 equations were also applied.^19^ Details of kidney function measurements are provided in appendix 5 (p 1).

### Statistical analysis

Continuous data were presented as median (IQR) and analysed using Wilcoxon rank-sum tests. Categorical data were analysed using Pearson’s χ^2^ or Fisher’s exact tests. Mortality analyses were performed on the pooled sample using Cox proportional hazards regression with age as the timescale and left truncation at each child’s age at enrolment to account for different start dates, enrolment ages, and follow-up durations across the two studies. Using age as the timescale aligned follow-up with biological age rather than calendar time, thereby accounting for age-related differences in baseline mortality risk and mitigating survival bias by ensuring comparisons were not confounded by differences in age at enrolment or length of follow-up across the two cohorts. Kaplan–Meier survival curves showed survival probabilities by AKI over time since enrolment (stratified by cohort) and participant age (pooled cohorts), with differences estimated using log-rank tests. Restricted mean survival time up to age 10 years was calculated according to AKI status. Changes in eGFR over follow-up were evaluated using linear mixed-effects regression with random intercept and slope and lagged between-visit dropout indicators capturing death or loss to follow-up to mitigate attrition bias. Odds of CKD and long-term major adverse kidney events were estimated using logistic regression, and incidences of all-cause hospitalisation were calculated with Poisson regression. Adjusted analyses considered the following enrolment characteristics: age, sex, height-for-age *Z* score, study site, study cohort, severe malaria group (ie, cerebral malaria, severe malarial anaemia, respiratory distress, complicated seizures, or prostration), and HIV status. Outcomes according to cohort, site, or sex were interpreted using the estimated results for these covariates from the appropriate regressions. Missing covariate data were minimal (2·6% missing HIV status), with results robust when including missing data as a category. p values less than 0·05 were considered significant, with Bonferroni-corrected thresholds applied for multiple testing where noted in figure descriptions. The present study followed STROBE reporting guidelines for cohort studies. Additional details on statistical analysis are provided in appendix 5 (p 2). Data were analysed using Stata version 18·0 (StataCorp) and R version 4·4·3 (R Foundation).

### Role of the funding source

The funders had no role in data collection, analysis, interpretation, writing, or the decision to submit.

## Results

After pooling the previous cohorts, 1077 children with severe malaria had enrolment creatinine concentrations and were included in the present analysis (figure 1; appendix 5 p 6). Enrolment characteristics of the study population combined and according to AKI are presented in the table. At enrolment, the median age of children was 2·5 years (IQR 1·8–3·5), 622 (57·8%) of 1077 were male, and 455 (42·2%) were female (table). The prevalence of AKI using creatinine was 40·0% (431 of 1077). The median length of follow-up was 6·5 years (3·7–8·8), and 686 (63·7%) of children were enrolled in the follow-up study. Overall, 147 (13·6%) died, and 56 (5·2%) withdrew or were lost to follow-up during the original study period (figure 1). Among children who completed the initial study, 175 (16·2%) were uncontactable and 13 (1·2%) declined participation in the follow-up study. Baseline characteristics according to loss to follow-up are presented in appendix 5 (p 7). Among participants re-enrolled in the follow-up study, 243 (35·4%) of 686 participants had AKI at the initial study enrolment. Participant age at the follow-up study timepoint ranged from 6 to 22 years (median 10 years [IQR 8·0–14·2]).

At enrolment, unadjusted prevalence of low eGFR was higher in children with AKI (431 [100%] of 431) compared with children without AKI (211 [32·7%] of 646; figure 2). Low eGFR at final follow-up remained more common in children with AKI (86 [37·9%] of 227) than in those without (124 [28·6%] of 433; p=0·015). Adjusted trajectories of eGFR showed that differences in mean eGFR by AKI status were largest at enrolment and reduced over time, with trajectories converging after the first year of follow-up (figure 2). The adjusted mean difference in eGFR at the final follow-up visit between children with and without AKI was −4·5 mL/min per 1·73 m^2^ (95% CI −8·5 to −0·45; p=0·029), but the difference was not significant after correction for multiple testing. Descriptively, serial eGFR values were consistently lower among children who died within one year of enrolment compared with those who survived (appendix 5 p 8).

Long-term major adverse kidney events occurred more frequently in children with AKI at enrolment compared with those without (137 [31·8%] of 431 *vs* 76 [11·8%] of 646, p<0·0001; figure 2), corresponding to a three-fold increase in the odds of long-term major adverse kidney events after adjustment (adjusted odds ratio [aOR] 3·14, 95% CI 2·23–4·43; p<0·0001). Among children with at least two post-discharge eGFR measurements 90 days apart, 68 (9·5%) of 713 met criteria for CKD. CKD was more common in children with AKI at enrolment compared with those without (33 [12·8%] of 257 *vs* 35 [7·7%] of 456, p=0·024; odds ratio 1·77, 95% CI 1·07–2·93; p=0·026), but this difference was not significant after adjustment for confounders (aOR 1·47, 95% CI 0·84–2·56; p=0·18). Adjusted odds of long-term major adverse kidney events and CKD were similar across cohorts but larger in participants enrolled in Jinja versus Kampala and in females versus males (appendix 5 p 9).

In the 656 (95·6%) of 686 participants with eGFR and urine albumin-to-creatinine ratio measurements at the final visit, the prevalence of kidney dysfunction was 20·7% (136 of 656) and did not differ between those with or without AKI at enrolment (47 [20·8%] of 226 *vs* 89 [20·7%] of 430, p=0·98; figure 2). Despite children with AKI at enrolment having lower unadjusted eGFR at the final follow-up visit compared with children without, urine albumin-to-creatinine ratio staging was similar by AKI status (p=0·99). When using eGFR equations that considered only cystatin C or combined creatinine-cystatin C, the distributions of CKD risk categories and kidney dysfunction were largely unchanged (appendix 5 p 10).

The all-cause mortality rate over follow-up was 13·6% (147 of 1077). Acute in-hospital deaths comprised 51·7% of the observed mortality (76 of 147), while the remaining 48·3% (71 of 147) occurred after discharge from the initial hospitalisation (median days to discharge 4 [IQR 3–6]). Of the 71 deaths that occurred post-discharge, 41 (57·7%) occurred at least 1 year after enrolment and 37 (52·1%) were recorded after the child’s completion of their previous study. The all-cause mortality rate was higher in children with AKI at enrolment than in children without (105 [24·4%] of 431 *vs* 42 [6·5%] of 646, p<0·0001), and this difference was evident for both in-hospital mortality (57 [13·2%] of 431 *vs* 19 [2·9%] of 646, p<0·0001) and post-discharge mortality (48 [12·8%] of 374 *vs* 23 [3·7%] of 627, p<0·0001; figure 3).

Across follow-up, AKI on admission conferred an over three-fold increase in the hazard of all-cause mortality (adjusted hazard ratio 3·36 [95% CI 2·22–5·10], p<0·0001; figure 3). To assess whether this elevated risk was confined to the short-term or persisted over the long-term, we did a series of Cox proportional hazards models conditioned on survival to landmark timepoints. Among children who survived the initial hospitalisation, the hazard of subsequent death remained higher in those with AKI (3·10 [1·83–5·27], p<0·0001). The risk persisted among those who survived at least one year (3·51 [1·75–7·03], p=0·0004) and remained elevated even among those surviving two or more years after the initial illness (4·53 [1·80–11·37], p=0·0013; figure 3).

Kaplan–Meier curves stratified by cohort showed early and sustained differences in survival probability by AKI status, with divergence occurring during the acute phase and widening over follow-up, particularly in cohort 2 (appendix 5 p 11). The adjusted hazards of death were also higher in cohort 2 versus cohort 1 and in Jinja versus Kampala, but similar in females versus males (appendix 5 p 9). In the pooled analysis, the greatest separation in survival probability by AKI status occurred at younger ages, consistent with the steep early divergence observed in both cohorts. After age 5 years, the gap began to stabilise, and at age 10 years, after which few deaths occurred, the cumulative probability of death among children with AKI reached 40%, compared with 12% for children without AKI (appendix 5 p 11). The restricted mean survival time up to age 10 years was 7·1 years (95% CI 6·5–7·7) for children with AKI compared with 9·2 years (8·9–9·5) for children without AKI (p<0·0001).

The incidence of all-cause hospitalisations after discharge was 117·8 per 100 person-years in children with AKI at enrolment, compared with 66·5 per 100 person-years in those without AKI. After adjustment for confounders, the incidence of readmission remained higher among children with AKI (adjusted incidence rate ratio 1·27 [95% CI 1·10–1·48], p=0·0013). Adjusted incidence rates of readmission were higher in cohort 2 versus cohort 1 and in Jinja versus Kampala but comparable between females and males (appendix 5 p 9).

## Discussion

AKI poses a major global health challenge, particularly in LMICs, where the burden of severe infection is high and health-care resources are scarce. In these settings, AKI frequently goes unrecognised and occurs in the context of life-threatening illnesses such as malaria.^7,20^ Although malaria-associated AKI has been consistently linked to increased in-hospital and short-term post-discharge morbidity and mortality in LMICs,^4–6,21^ most prospective paediatric studies have followed up children for 1 year or less, limiting the understanding of whether AKI puts children at sustained risk of death or progressive kidney disease years after the initial illness.

In this prospective cohort of 1077 Ugandan children hospitalised with severe malaria and followed up for up to 15 years, AKI on admission was strongly associated with excess long-term mortality, with children with AKI experiencing an average of 2·1 fewer years of survival up to age 10 years than those without AKI. Although children with AKI on admission were more likely to develop CKD over follow-up, this association was attenuated after adjustment, and differences in eGFR by AKI status diminished over time among survivors. Mortality was therefore the primary driver of long-term major adverse kidney events, with children who developed AKI experiencing more than a three-fold increase in the hazard of death that persisted beyond 2 years after the initial illness. Despite scarce data on outcomes following paediatric AKI in LMICs beyond 1 year, our findings are broadly consistent with a multicentre cohort from India reporting high mortality and frequent non-recovery of kidney function in children with AKI.^22^ In contrast to that study, kidney function among survivors in our study largely recovered. Greater use of kidney replacement therapy in the Indian cohort and differences in case mix and pre-existing kidney disease might partly explain this divergence, as children with more severe or non-recovering AKI in our setting might have died before CKD could be observed. In our study, serial eGFR measurements during the first year of follow-up were lower among children who subsequently died than those who survived, underscoring the close link between early kidney dysfunction and mortality. Taken together, these data support the hypothesis that, in LMICs, elevated mortality over time could limit the detection of long-term kidney dysfunction following paediatric AKI. These findings contrast with evidence from high-income settings, which show AKI is associated with adverse long-term outcomes, including CKD, recurrent hospitalisation, and cardiovascular morbidity, with relatively low mortality.^9,23,24^

The predominance of mortality over CKD as the driver of long-term major adverse kidney events in this population is likely attributable to systemic and biological factors that distinguish resource-limited, malaria-endemic settings from high-income contexts. Limited laboratory capacity for early AKI recognition, restricted access to kidney replacement therapy and paediatric nephrology care, and constrained post-discharge follow-up likely contribute to worse outcomes after AKI in LMICs such as Uganda.^25,26^ Our findings are consistent with a multicentre cohort study of critically ill adults with severe AKI in low-resource settings in India and southeast Asia that reported mortality as the dominant component of major adverse kidney events at 2 years.^27^ AKI in LMICs also frequently occurs in the context of severe infection. Malaria-associated AKI is characterised by systemic inflammation, haemolysis, and multiorgan dysfunction,^21^ and recurrent malaria episodes—each carrying a risk of AKI—could compound long-term mortality risk. Consistent with this vulnerability, children with AKI in our cohort experienced higher rates of all-cause hospitalisation during follow-up than those without AKI. Fewer systemic infections and more consistent follow-up care in high-income settings, compared with low-income settings, enable survival after AKI and allow progression to CKD and other sequelae to be identified and treated.^8,24,25^

Mortality following AKI was higher in cohort 2 than cohort 1, likely reflecting differences in cohort composition or study timing. Cohort 2 was younger (ie, aged <5 years at enrolment, the age group with the highest burden of severe malaria-related mortality),^1^ included severe malaria manifestations associated with greater mortality risk (eg, respiratory distress),^28^ and additionally enrolled children from Jinja, a rural site with moderate-to-high malaria transmission (compared with peri-urban Kampala with low transmission).^29^ Consistent with these cohort characteristics, children in Jinja had higher incidence of all-cause hospitalisation, greater odds of CKD and long-term major adverse kidney events, and increased hazard of mortality compared with those in Kampala. However, even after accounting for age, severe malaria group, and study site, mortality risk remained higher in cohort 2, potentially reflecting unmeasured differences in underlying population risk or temporal changes in epidemiology between study periods, which warrant further investigation. Sex differences were less consistent, with higher CKD among female participants but no corresponding difference in mortality or hospitalisation risk during follow up.

Strengths of this study include the long follow-up period, which enabled assessment of mortality and CKD beyond the acute phase of AKI, as well as the prospective design and large sample size. However, our study has several limitations. First, loss to follow-up occurred primarily between completion of the previous studies and enrolment in the follow-up study and could have introduced selection bias. Baseline characteristics were similar between children who were retained versus children who were lost to follow-up, although survivor bias remains possible. Loss to follow-up was less common among children with AKI compared with those without, and we used modelling approaches that accounted for death and loss to follow-up in longitudinal kidney function analyses, including joint modelling (appendix 5 p 12), with consistent results across methods. Second, there was an age imbalance between AKI groups with children with AKI younger than those without, and despite adjustment, residual unmeasured heterogeneity remains possible. Our findings should not be interpreted as causal, and future research should incorporate complementary approaches to strengthen causal inference. Third, we imputed exit ages for children lost to follow-up, although results were robust to alternative imputation strategies (appendix 5 p 13). Fourth, because creatinine was measured only at scheduled visits, we were unable to assess recurrent AKI events, despite evidence from high-income settings that recurrent AKI contributes to long-term mortality,^30^ or to characterise the precise timing and trajectory of CKD development. Fifth, reliance on serum creatinine and cystatin C might not fully capture kidney dysfunction following malaria-associated AKI, and additional studies assessing total urine protein measures and tubular biomarkers are needed. Sixth, an absence of imaging to exclude congenital anomalies of the kidney urinary tract limit our ability to assess pre-existing kidney disease. We also did not fully adjudicate alternative causes of AKI, such as sepsis, although bacteraemia prevalence was low (5%) and not associated with AKI at enrolment. Future studies in low-resource settings should take a multifactorial approach to AKI phenotyping and outcome assessment to better define mechanisms linking early kidney injury and long-term mortality.

In the present study, we report increased long-term major adverse kidney events in children following severe malaria-associated AKI with sustained long-term risk of mortality and a higher incidence of hospitalisation within 1 year of discharge compared with children without AKI on admission for severe malaria. Our findings underscore the need to reimagine AKI care in LMICs, with a focus on preventing both short-term and long-term mortality. Unlike high-income settings, where advances in AKI risk stratification and longitudinal care have improved survival, AKI management in resource-limited settings remains largely supportive, with little access to early diagnostic testing, nephrology care, and post-discharge follow up.^24–26^ The stark contrast in mortality outcomes suggests that many deaths following malaria-associated AKI could be preventable. Health systems in malaria-endemic regions should prioritise early AKI recognition in high-risk populations, strengthen referral pathways and post-discharge surveillance, and integrate kidney health into existing child survival and malaria programmes.^31^ Recognising AKI as a sentinel event rather than a transient complication is essential to reducing mortality among children surviving severe malaria.

### Equitable Partnership Declaration

The authors of this paper have submitted an Equitable Partnership Declaration (appendix 6). This statement allows researchers to describe how their work engages with researchers, communities, and environments in the countries of study. This statement is part of The Lancet Group’s broader goal to decolonise global health.

## Supplementary Material

1

2

3

4

5

6

## Figures and Tables

**Figure 1:**
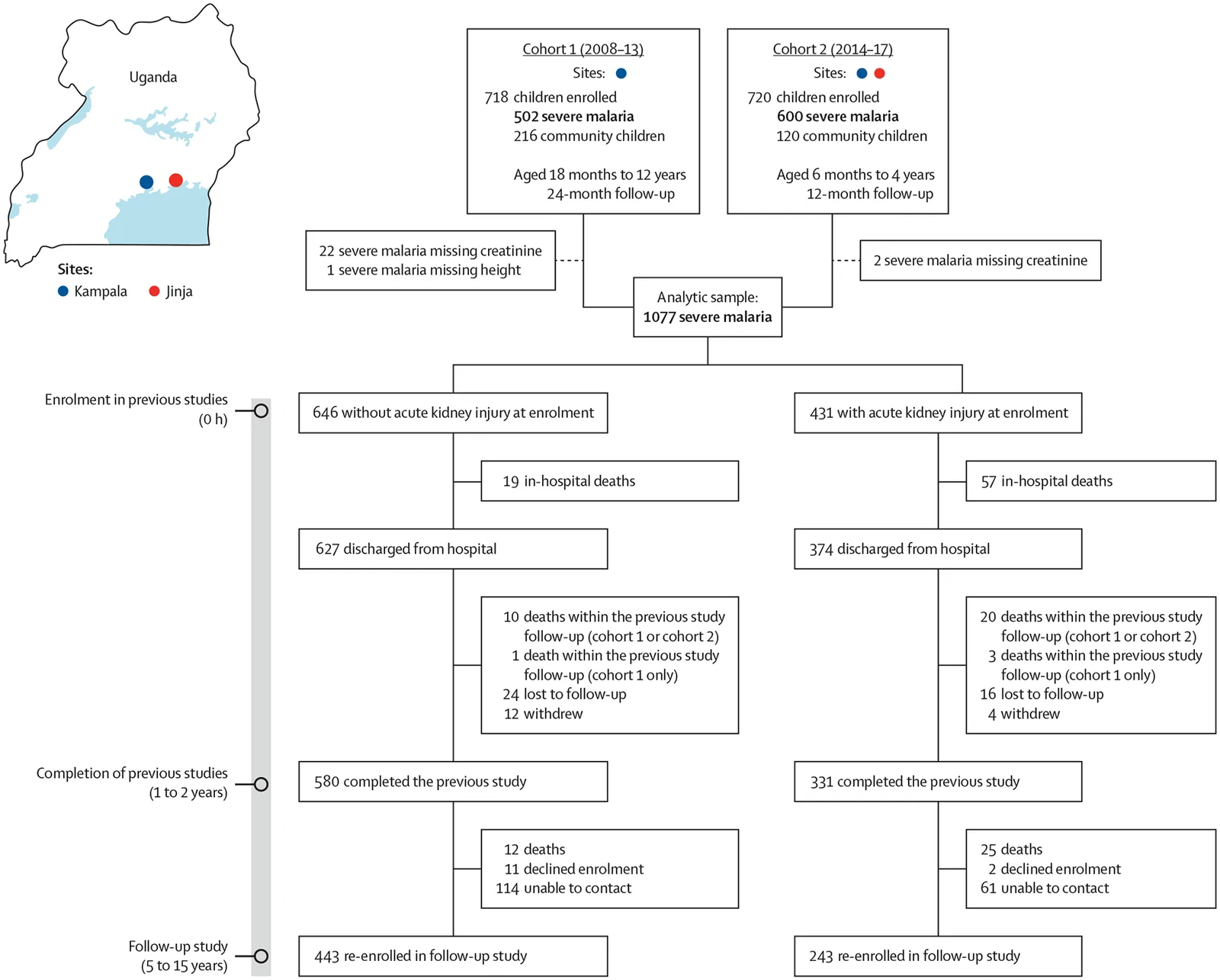
Flowchart of study enrolment and follow-up The selection and retention from two prospective study cohorts of Ugandan children with severe malaria, pooled for the present analysis, is shown. The figure includes the number of children surviving to key study timepoints, stratified by the presence or absence of acute kidney injury at previous study enrolment.

**Figure 2:**
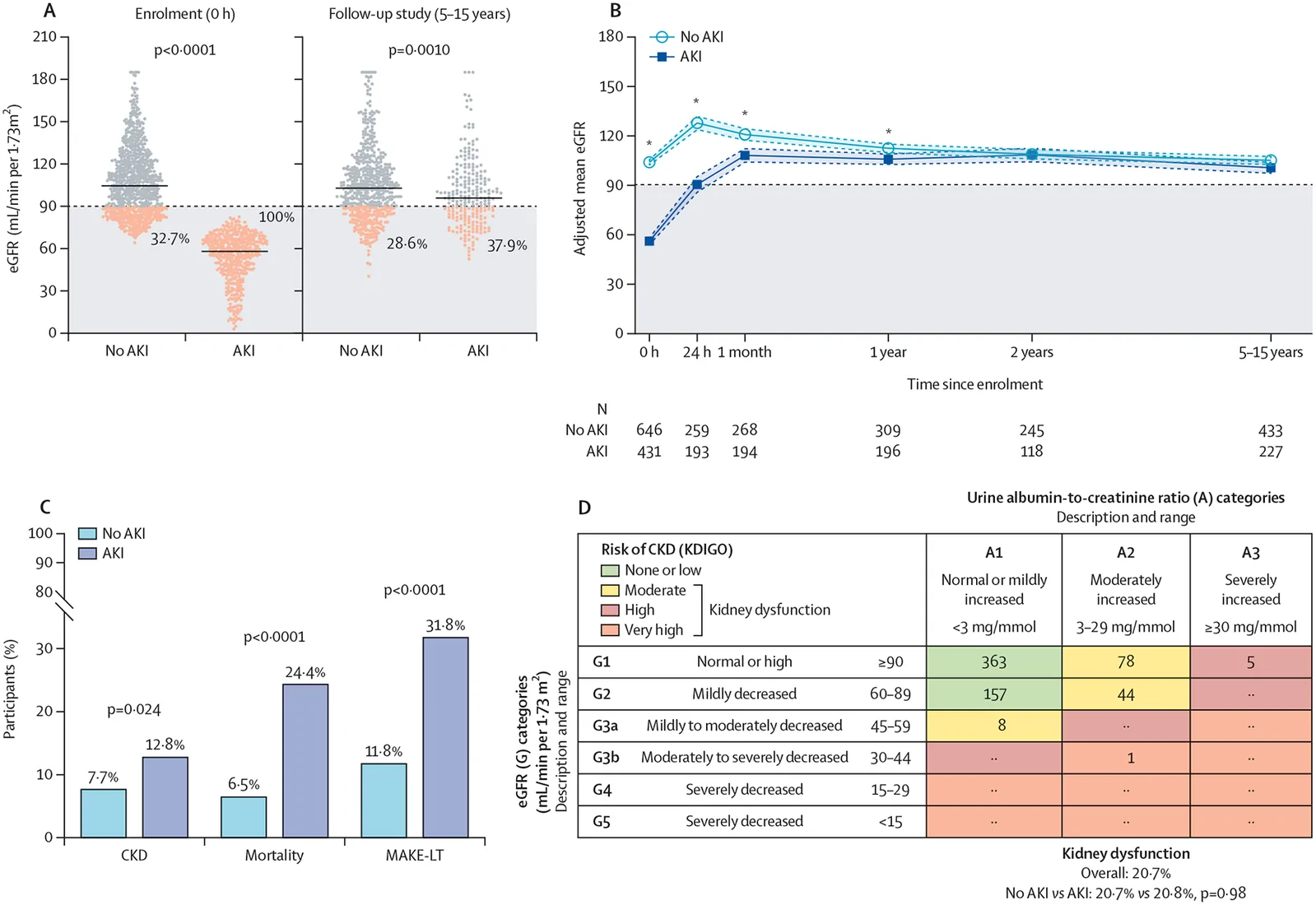
Long-term kidney outcomes according to AKI on admission for severe malaria (A) Dot plots with median bars show eGFR at enrolment of the previous study and at enrolment of the follow-up study, stratified by AKI status at enrolment of the previous study. Grey shading and orange dots below the dashed line indicate low eGFR (<90 mL/min per 1·73 m^2^). eGFR estimated using the Chronic Kidney Disease in Children Under 25 equation. p values were obtained from Wilcoxon rank-sum tests. (B) Marginal predicted eGFR at scheduled testing timepoints according to AKI status at previous study enrolment. Estimates were derived from a linear mixed-effects model adjusted for age, sex, height-for-age *Z* score, study site, study cohort, severe malaria group, and HIV status, with a subject-specific intercept and random slope. Testing timepoints and between-timepoint dropout indicators were modelled as categorical variables. The dotted line shows normal eGFR at 90 mL/min per 1·73 m^2^. The dotted lines around the datapoints represent 95% CIs. Grey shading below the dashed line indicates low eGFR (<90 mL/min per 1·73 m^2^), on average. (C) Bar charts show CKD, mortality, or MAKE-LT over follow-up by AKI status on hospital admission. p values derived from χ^2^ tests. (D) Number of children in each CKD risk category based on KDIGO consensus criteria at the final follow-up timepoint, determined by the combination of eGFR and urine albumin-to-creatinine ratio categories. Persistent kidney dysfunction among children enrolled in the follow-up study was defined as a moderately increased or greater risk of CKD (ie, eGFR <60 mL/min per 1·73 m^2^ or urine albumin-to-creatinine ratio ≥3 mg/mmol). AKI=acute kidney injury. CKD=chronic kidney disease. eGFR=estimated glomerular filtration rate. KDIGO=Kidney Disease: Improving Global Outcomes. MAKE-LT=long-term major adverse kidney events. *Denotes significant differences in eGFR at a given timepoint after Bonferroni correction for six timepoints (p<0·0083).

**Figure 3:**
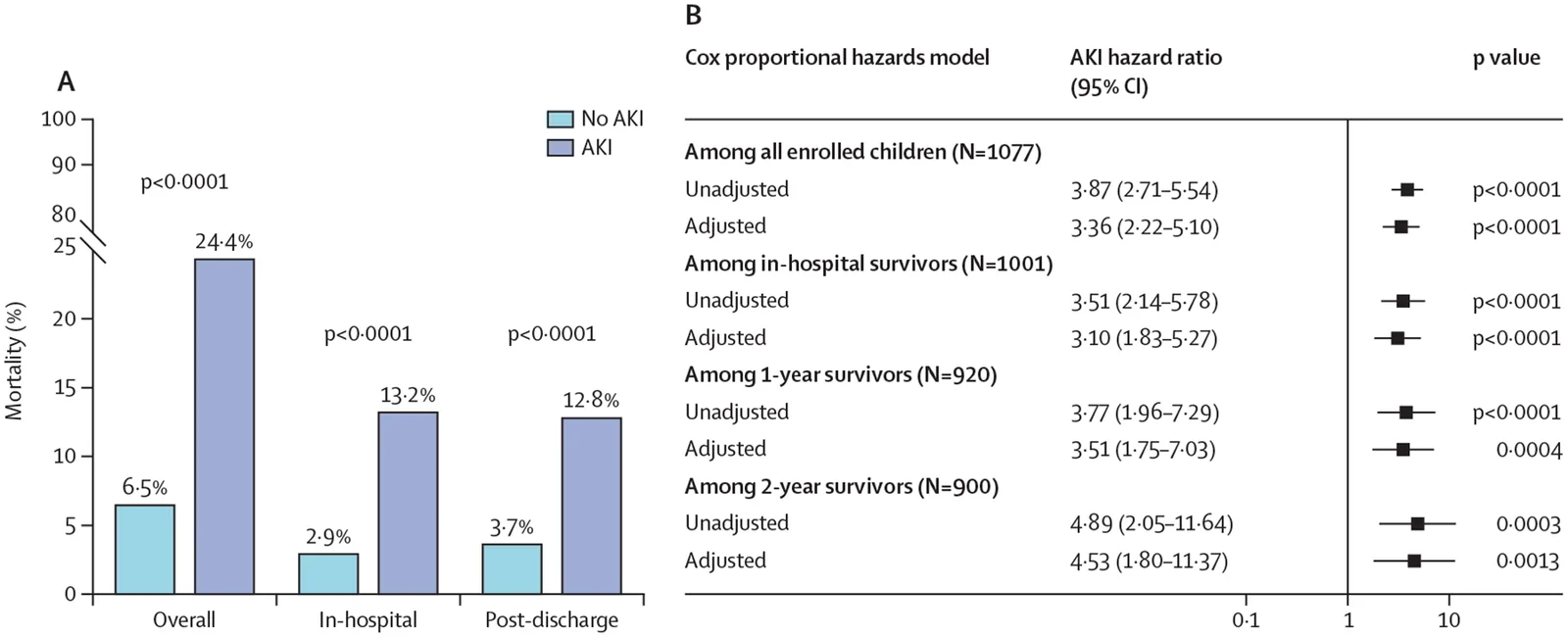
Mortality over follow-up according to AKI on admission for severe malaria (A) Bar charts show in-hospital, post-discharge, and overall mortality by AKI status on admission. p values derived from χ^2^ tests. (B) Forest plot showing hazard ratios and 95% CIs from four sets of Cox proportional hazards regression models of death with age as the timescale: (1) among all enrolled children, (2) among survivors of the initial severe malaria hospitalisation, (3) among children who survived 1 year post-enrolment, and (4) among children who survived 2 years post-enrolment. Hazard ratios are presented both unadjusted and adjusted for enrolment characteristics, including sex, height-for-age *Z* score, study site, study cohort, severe malaria group, and HIV status. AKI=acute kidney injury.

**Table:** Baseline demographics, clinical characteristics, and laboratory characteristics of children according to acute kidney injury on hospital admission for severe malaria

	N	No acute kidney injury	N	Acute kidney injury	p value
**Demographics**					
Age, years	646	2·6 (1·9 to 3·5)	431	2·4 (1·7 to 3·4)	0·0081
Sex	646	··	431	··	0·62
Male	··	377 (58·4%)	··	245 (56·8%)	··
Female	··	269 (41·6%)	··	186 (43·2%)	··
Cohort	646	··	431	··	0·0030
1	··	335 (51·9%)	··	263 (61·0%)	··
2	··	311 (48·1%)	··	168 (39·0%)	··
Site	646	··	431	··	<0·0001
Kampala	··	530 (82·0%)	··	279 (64·7%)	··
Jinja	··	116 (18·0%)	··	152 (35·3%)	··
Weight-for-age *Z* score	642	−1·0 (−1·8 to −0·3)	424	−1·1 (−1·8 to −0·4)	0·38
Height-for-age *Z* score	646	−1·1 (−2·0 to −0·2)	431	−1·1 (−1·9 to −0·4)	0·92
**Clinical characteristics**					
Duration of fever, days	641	3 (2 to 5)	425	3 (2 to 4)	<0–0001
Axillary temperature, °C	646	37·7 (36·9 to 38·5)	431	37·7 (36·9 to 38·5)	0·98
Coma	646	179 (27·7%)	431	166 (38·5%)	0·0002
Multiple seizures	644	113 (17·5%)	429	114 (26·6%)	0·0004
Respiratory distress	646	70 (10·8%)	431	140 (32·5%)	<0·0001
Blackwater fever	644	97 (15·1%)	430	127 (29·5%)	<0·0001
Severe anaemia	645	315 (48·8%)	429	205 (47·8%)	0·74
Prostration	646	424 (65·6%)	431	366 (84·9%)	<0·0001
Jaundice	646	236 (36·5%)	430	188 (43·7%)	0·018
HIV infection	632	15 (2·4%)	417	9 (2·2%)	0·82
**Laboratory findings**					
White blood cells, ×10^3^/μL	642	10·7 (7·7 to 15·7)	424	12·6 (8·9 to 20·8)	<0·0001
Platelet count, ×10^3^/μL	643	108 (61 to 203)	424	95 (47 to 191)	0·022
Haemoglobin, g/dL	645	5·1 (3·9 to 8·0)	429	5·1 (3·6 to 7·7)	0·56
Parasite density, parasites per μL	643	32 088 (2170 to 167389)	424	27632 (1102 to 207447)	0·92
Plasma histidine-rich protein 2, ng/mL	641	1685 (418 to 3930)	430	3184 (765 to 6794)	<0·0001
Bacteraemia	600	28 (4·7%)	411	22 (5·4%)	0·62
Lactate, mmol/L	605	3·7 (2·1 to 6·2)	412	5·2 (3·0 to 10·3)	<0·0001
Blood urea nitrogen, mg/dL	642	11 (7 to 15)	430	22 (15 to 34)	<0·0001
Creatinine, mg/dL	646	0·31 (0·26 to 0·36)	431	0·52 (0·45 to 0·70)	<0·0001

Data are n (%) or median (IQR). p values obtained from Wilcoxon rank–sum tests for continuous variables and Pearson’s *χ*^*2*^ tests for categorical variables.

## Data Availability

The datasets used for the primary analyses will be made available in a GitHub repository with publication, including de-identified participant data and a data dictionary. Data can be used for research purposes without investigator support, provided that appropriate citation of this Article is included.

## Research in context

### Evidence before this study

We searched PubMed for studies published in English from database inception to April 1, 2026, using combinations of the search terms “acute kidney injury”, “malaria”, “paediatric”, “children”, “long-term outcomes”, “mortality”, and “chronic kidney disease”. Reference lists of relevant articles were also screened. Acute kidney injury (AKI) occurs in nearly one-third of children admitted to or treated within hospital globally, with most cases occurring in low-income and middle-income countries (LMICs), where infections are a major driver. Malaria is a leading cause of AKI, which affects up to 60% of children with severe malaria. Existing cohort studies, primarily with follow-up of 1 to 2 years, show that concomitant severe malaria and AKI is associated with increased mortality, chronic kidney disease, and neurocognitive impairment compared with severe malaria without AKI. In contrast, paediatric studies in high-income settings with longer follow-up have reported an association between AKI and chronic kidney disease, hospital admission, and hypertension, but low mortality. Comparable long-term follow-up data from children in LMICs are scarce, particularly beyond the first 1 or 2 years after AKI.

### Added value of this study

This study addresses the scarcity of long-term data on paediatric AKI outcomes in LMICs by evaluating survival and kidney outcomes in more than 1000 Ugandan children admitted to hospital with severe malaria followed for up to 15 years (median 6·5 [IQR 3·7–8·8]). By examining long-term major adverse kidney events following paediatric AKI in a malaria-endemic, low-income setting, this study shows that the long-term consequences of AKI differ from those observed in high-income settings, with excess mortality rather than chronic kidney disease predominating.

### Implications of all the available evidence

AKI is increasingly recognised as an event with consequences extending beyond the acute illness. In LMICs, where severe infections of any bacteria or virus are common and access to follow-up care is scarce, the long-term impact of AKI appears to be driven primarily by excess mortality rather than chronic kidney disease. These results suggest that paediatric AKI in LMICs could serve as a sentinel event identifying children at prolonged risk of death and underscore the need to strengthen early recognition, post-discharge surveillance, and integrated child survival care in resource-limited settings.
