# Supplementary material for: Long-term mortality and risk of chronic kidney disease in children following severe malaria complicated by acute kidney injury: a prospective observational study
Source: Lancet Glob Health. Author manuscript; Available in PMC 2026 Nov 2. (PMC13726406; doi:10.1016/j.langlo.2026.103984)
Supplement: Supplementary file 1 [file NIHMS2215924-supplement-1.pdf]

THE LANCET
Global Health

Supplementary appendix 1

This translation in Swahili was submitted by the authors and we reproduce it as supplied. It has not been peer reviewed. *The Lancet*'s editorial processes have only been applied to the original in English, which should serve as reference for this manuscript.

Tafsiri hii katika Swahili iliwasilishwa na waandishi na tunatengeneza tena kama hutolewa. Haijapitiwa. Mchakato wa hariri wa Lancet umetumika tu kwa asili kwa Kiingereza, ambayo inapaswa kutumika kama kumbukumbu kwa muswada hii.

Supplement to: Mellencamp KA, Namazzi R, Batte A, et al. Long-term mortality and risk of chronic kidney disease in children following severe malaria complicated by acute kidney injury: a prospective observational study. *Lancet Glob Health* 2026. https://doi.org/10.1016/j.langlo.2026.103984

Swahili Summary

Muhtasari

Usuli. Jeraha kali la figo maambukizi ya ghafla ya figo ni tatizo linaloweza kutokea kwa watoto wenye malaria kali, lakini matokeo yake ya muda mrefu bado hayajaeleweka vizuri.kwa nchi zenye kipato cha chini na cha kati. Tulilenga kuchunguza uhusiano wa muda mrefu kati ya watoto na hali ya figo pamoja na vifo baada ya malaria kali.

Mbinu. Tulichanganya takwimu kutoka tafiti mbili za kufuatilia watoto wa Uganda (wenye umri wa miezi 6 hadi miaka 12) waliolazwa hospitalini kwa malaria kali kati ya mwaka 2008 na 2017. Watoto waliokuwa na sampuli za damu zilizohifadhiwa wakati wa kulazwa hospitalini kwa ajili ya kupima kreatini walijumuishwa katika uchambuzi. Walionusurika waliingizwa katika utafiti wa ufuatiliaji uliofanyika kati ya 2020 hadi 2023, ambapo hali ya figo na uhai vilipimwa. Mbinu ya takwimu ya uchanganuzi wa cox ilitumika kuchanganua hatari ya vifo, hukuuchanganuzi WA urejeleaji wa lojestiki ikitumika kukadiria uwezekano wa ugonjwa sugu wa figo na matukio makubwa ya muda mrefu ya figo.Uchambuzi uliorekebishwa ulijumuisha sifa zifuatazo za waliohusishwa kwenye utafiti:umri,jinsia,kiwango cha urefu kulingana na umri(eneo la utafiti,kundi la malaria kali yaani malaria ya ubongo,upungufu mkubwawa damu unaosababishwa na malaria,matatizo ya kupumua,degedege kali au hali ya kulegea sana mwilini,pamoja na hali ya maambukizi ya vvu.

Matokeo. Wakati wa kuanza utafiti, wastani wa umri ulikuwa miaka 2.5, ambapo asilimia 57.8 walikuwa wavulana na asilimia 42.2 wasichana. Jeraha kali la figo ilipatikana kwa asilimia 40 ya watoto. Katika kipindi cha ufuatiliaji cha takriban miaka 6.5, asilimia 13.6 ya watoto walifariki, na karibu nusu ya vifo vilitokea baada ya kuruhusiwa kutoka hospitalini. Watoto waliopata jeraha kali la figo walikuwa na uwezekano mkubwa wa kupata matokeo mabaya ya muda mrefu ya figo na hatari kubwa ya vifo, hata zaidi ya miaka miwili baada ya kuugua. Pia walionekana kuwa na uwezekano mkubwa wa kupata ugonjwa sugu wa figo, ingawa baada ya marekebisho ya takwimu, matokeo hayo hayakuwa na uthibitisho mkubwa.

Ufafanuzi. Katika kundi hili la watoto, jeraha kali la figo ilihusishwa na ongezeko la vifo vya muda mrefu. Hii inaonyesha kuwa jeraha kali la figo ni ishara muhimu ya hatari ya kudumu ya vifo na inaonyesha udhaifu wa mifumo ya huduma za afya zinazolenga zaidi matibabu ya muda mfupi katika maeneo yenye malaria nyingi.

Maneno Muhimu. Jeraha kali la figo; malaria kali; uhai wa mtoto; ugonjwa sugu wa figo; matokeo ya muda mrefu; nchi zenye kipato cha chini.

Tafsiri ya lugha ya kiswahili iliwasilishwa na waandishi, nasi tumeichapisha kama ilivyowasilishwa. Haijapitiwa na wataalamu wengine kwa ajili ya uhakiki. Taratibu zilizofuatwa na mchapishaji,kwa Lancet, katika kupitia, kuboresha na kuandaa muswada zilihusu tu nakala ya awali ya Kiingereza, ambayo ndiyo inapaswa kutumika kama rejea ya msingi kwa muswada huu.
