# Supplementary material for: Long-term mortality and risk of chronic kidney disease in children following severe malaria complicated by acute kidney injury: a prospective observational study
Source: Lancet Glob Health. Author manuscript; Available in PMC 2026 Nov 2. (PMC13726406; doi:10.1016/j.langlo.2026.103984)
Supplement: Supplementary file 2 [file NIHMS2215924-supplement-2.pdf]

THE LANCET
Global Health

Supplementary appendix 2

This translation in Luganda was submitted by the authors and we reproduce it as supplied. It has not been peer reviewed. *The Lancet's* editorial processes have only been applied to the original in English, which should serve as reference for this manuscript.

Ekiwandiiko kino ekikyuse mu lulimi Oluganda kyaweereddwayo abawandiisi b'ennyini era tukifulumizza nga bwe baakituwadde. Tekinnakubaganyizibwako birowoozo abakugu abalala. Okusunsula kwa Lancet kukoleddwa ku ekyo eky'Olungereza kyokka nga bwe kyafulumizibwa era kye kirina okujulizibwako ku kiwandiiko kino.

Supplement to: Mellencamp KA, Namazzi R, Batte A, et al. Long-term mortality and risk of chronic kidney disease in children following severe malaria complicated by acute kidney injury: a prospective observational study. *Lancet Glob Health* 2026. https://doi.org/10.1016/j.langlo.2026.103984

Luganda Summary

Ebikwata ku kunoonyereza mu bufunze

Ennyanjula. Obukosefu bw'ensigo obwembagirawo mu baana kizibu ekireetebwa omusujja oguva ku nsiri ogwamaanyi. Wabula, mu mawanga amaavu era agakyaakula, ebiva mu bukosefu buno oluvanyuma nga wayise ebbanga tebinnyonnyolwa bulungi. Kye twagenderera mu kukola okunoonyereza kuno kwaali okwetegereza akakwate akaliwo oluvanyuma nga wayise ebbanga wakati w'ebiva mu kukosebwa kw'ensigo kuno n'omuwendo gw'abaana abafa oluvanyuma lw'okulwala omusujja oguleetebwa ensiri ogwamaanyi.

Engeri okunoonyereza gye kwakolebwamu. Wakati w'omwaka gwa 2008 n'omwaka ogwa 2017, twakung'aanya era ne tugatta wamu ebikwata ku bibiinja by'abaana bannayuganda bibiri (abaali baweza ebbanga eriri wakati w'emyezi mukaaga n'emyaka kkumi n'ebiri), era abaali baweereddwa ebitanda mu malwaliro nga balina omusujja oguleetebwa ensiri ogwamaanyi. Abaana abeekeenenyezebwa beebo abaali baggyiddwako omusaayi mu kiseera webaabeerera ku kitanda olw'okugukebera engeri ensigo zaabwe gyezikolamu Abaana abaali bakyaali abalamu bawandiisibwa okwetaba mu kunoonyereza okwalimu okulondoolebwa okwakolebwa wakati w'omwaka 2020 ne 2023-ekiseera okukebera enkola y'ensigo zaabwe ne bbanga lye bamala nga balamu we kwakolebwa nga tweyambisa enkola etunuulira engeri ebintu eby'enjawulo gyebikosaamu emyaka egiyitawo ng'omuntu tannaffa.. Enkola endala eyakozesebwa yeeyo eteebereza obulabe obuliwo okukwatibwa oba obutakwatibwa bulwadde bwa nsigo obw'olukonvuba(ng'ebiva mu kukebera enkola y'ensigo okw'emirundi ebiri okukolebwa oluvanyuma lw'ennaku ezissuka mu 90 n'obulabe obuva mu kubeera obulwadde bw'ensigo nga wayise ebbanga kuba kufa oba kubeera na bulwadde bwa nsigo obw'olukonvuba biraga nti kuli wansi.. Ensonga endala ezaatunuulirwa era ezekeneenyezebwa mwe mwaali emyaka gy'omulwadde, ekikula kye, obuwanvu bw'omulwadde ng'obugerageranyiza n'emyaka gye, ekifo okunoonyereza we kwakolerwa, ekibiinja ky'abalwadde abaanoonyerezebwako, ekibiinja ky'abalwadde b'omusujja oguleetebwa ensiri(okugeza omusujja gw'ensiri ogukosa obwongo, obutaba na musaayi gumala, obuzibu mu kussa, okwesika okubi ennyo, okunafuwa ennyo) n'okuba oba omulwadde yalina akawuka ka siliimu oba nedda.

Ebyaazuulibwa mu kunoonyereza. Mu kuwandiisibwa okwetaba mu kunoonyereza, emyaka gy'abaana abasinga obungi gyaali ebiri n'ekitundu 2.5 (1.8‒3.5). Ebitundu 57.8% ku baana abeetaba mu kunoonyereza (622/1077) baali balenzi, ebitundu 42.2% baali bawala, nga naabo abaalina obukosefu bw'ensigo obw'embagirawo baali ebitundu 40% (431/1077) ku muwendo gw'abaana bonna. Mu baana abalondoolwa okumala emyaka egiri wakati w'esatu n'emyaka munaana n'emyezi munaana (3.7‒8.8), abaana ebitundu 13.6% baafa, era nga kumpi kimu kyakubiri ku baana abaafa, baafa luvanyuma nga bamaze okusiibulwa okuva mu ddwaliro. Ng'oggyeeko ensonga endala, abaana abaalina obukosefu bw'ensigo obwembagirawo, ebitundu 95% baali mu kabi kanene okukwatibwa obulwadde bw'ensigo obw'olukonvuba oba okufa. Abaana abaasumatuka okufa obukosefu bw'ensigo, ebitundu 95% baali mu kabi kanene ak'okukwatibwa obulwadde bw'ensigo obw'olukonvuba.

Entaputa y'ebyaava mu kunoonyereza. Mu kibiinja ky'abaana bano abaanoonyerezebwako, kyaazuulibwa nti obukosefu bw'ensigo obwembagirawo butera nnyo okuvaamu okufa oluvanyuma nga wayise ebbanga, ekiraga nti obukosefu bw'ensigo obubi ennyo kabonero akatalaga kubaawo kwa buzibu bwokka, wabula n'akabi ak'okufa, era ekyongera okulaga obunafu obuli mu kujjanjaba endwadde embi ennyo mu malwaliro agali mu bifo omusangibwa ennyo omusujja oguleeta ensiri.

Ebigambo ebikulu. Obukosefu bw'ensigo obw'embagirawo; omusujja oguleetebwa ensiri ogwamaanyi; okuwangaala kw'abaana; obulwadde bw'ensigo obw'olukonvuba; ebiva mu kulwala nga wayise ebbanga; essente ezitamala.

Okuvvuunula kuno mu lulimi oluganda kwaweebwaayo bawandiisi era naffe tukukoppolola nga bwe kwatuweebwa. Tekwekeneenyezeddwa bakugu. Emitendera egigobererwa kampuni eyitibwa Lancet mu kwekebejja, okulongoosa n'okuteekateeka ebiwandiikiddwa gigobereddwa mu kkopi eyasooka yokka eri mu lulimi olungereza; era eyo y'erina okujulizibwa mu kiwandiiko kino.
