# Supplementary material for: Long-term mortality and risk of chronic kidney disease in children following severe malaria complicated by acute kidney injury: a prospective observational study
Source: Lancet Glob Health. Author manuscript; Available in PMC 2026 Nov 2. (PMC13726406; doi:10.1016/j.langlo.2026.103984)
Supplement: Supplementary file 3 [file NIHMS2215924-supplement-3.pdf]

THE LANCET
Global Health

Supplementary appendix 3

This translation in Lusoga was submitted by the authors and we reproduce it as supplied. Ithas not been peer reviewed. *The Lancet*' editorial processes have only been applied to theoriginal in English, which should serve as reference for this manuscript.

Okuvhuunula kunho mu lulimi olusoga kwaghebwaayo abaghandiisi era niiffe tukukoppolola nga bwe kwatughebwa. Ttikwekenhenhiezeibwa bakugu. Emitendera edhigobererwa kampuni eyetibwa Lancet mu kwekebeedha, okulongoosa n'okuteekateeka ebighandiikiibwa dhigobeleilwa mu kkopi eyasooka yonka eri mu lulimi olungereza; era eyo nerina okwidhulizibwa mu kiwandiiko kino.

Supplement to: Mellencamp KA, Namazzi R, Batte A, et al. Long-term mortality and risk of chronic kidney disease in children following severe malaria complicated by acute kidney injury: a prospective observational study. *Lancet Glob Health* 2026. https://doi.org/10.1016/j.langlo.2026.103984

Lusoga Summary

Ebigema ku kunhonhenkereza mu bufunze

Enandhula. Okukosebwa kw'ensigo okw'amangu kizibu ekiletebwa omusudha gw'ensiri ogw'amaanhi mu baana abato. Aye, ebiva mukyo oluvainhuma nga ghabise eibbanga ttibinhongolwa bukalamu mu maghanga ag'enfuna entono n'ago agaliku kalekale mu byenfuna. Kyetwagenderera mu kukola okunhonhenkereza kunho n'okwetegereza akakwate akaligho oluvainhuma nga wabise eibbanga wagati ghebiva mu kukosebwa kw'ensigo kunho n'omuwendo ogw'abaana abafa oluvainhuma lw'okulwala omusudha oguleetebwa ensiri ogwamaanhi.

Engeri okunhonhenkereza dhekwakolebwaamu. Twasolooza amawulire okuva mu bibindha bibiri eby'abaana ba Uganda (abali wagati w'emyezi 6 okutuuka ku myaka 12) abaghebwa ebitandha mu'ilwaliro nga balina omusudha gw'ensiri ogw'amaanhi wagati gh'omwaka 2008 nhi 2017. Abaana abaalina omusaayi ogwatelekebwa okukebera enkola y'ensigo byoona byatekebwa mu'ebyo ebyekenhezeibwa. Abaana abasigalagho nga balamu bayingizibwa mukunhonhenkereza okw'okulondoola okwakolebwa okuva mu 2020 okutuuka mu 2023, bwebakebela okukola kw'ensigo n'abo abawonagho, nga bagerageranhia emyaka edh'omwana n'obuzibu bw'okufa. Enkola eyakozesebwa okutebereza emikisa dh'okugemebwa obulwaire bw'ensigo obutaghona, ekitegerekeka ng'ebiva mu kukebera ensigo emirundi eibiri oba okusingagho edh'omuddiringanwa munnaku 90 oba okusingagho, n'ebibi ebiva mu kulwala kw'ensigo eby'ekiseera ekighanvu, ebimanikiibwa okulemelerwa kwensigo okukola okumala emyezi esatu oba okusingagho oba okufa . Okwekenenhiezebwa okwakyusibwa kwalimu bino ghamanga: emyaka, ekikula, obughanvu nga bugerageranhizibwa n'emyaka, aghakolebwa okunhonhenkereza, ekibiina eky'abantu ekikolebwaaku okunhonhenkereza, ekibiina ekilina omusudha ogw'amaanhi (okugeza, omusudha g'ensiri ogwomubwongo, omusudha gw'ensiri ogw'amaanhi ogumalamu omusaayi, okuzibughalirwa mu kwiisa omuuka, okukankana okw'amaanhi, oba okugheramu ilala amaanhi), n'embeera mu bulwaire obw'akawuuka ka silimu.

Ebyava mukunhonhenkereza. Mukwingizibwa mukunhonhenkereza, emyaka dh'abaana abasinga dhaali 2·5 (1·8–3·5), Ebitundhu 57·8% (622/1077) abetaba mukunhonhenkereza baali balenzi. Ebitundhu 42·2% (455/1077) baali bawala, nga nabo abaalina obukosefu obw'ensigo obw'amangu baali 40% (431/1077) ku mughendo gw'abaana gwona gwona. Mu baana abalondoolwa okumala emyaka edhiri wagati w'eisatu n'emyezi musanvu n'emyaka omunaana n'emyezi munaana (3.7–8.8), abaana ebitundhu 13.6% baafa, era nga kumpi kilala kyakubiri ku baana abaafa, baafa oluvainhuma nga bamaze okusiibulwa okuva mu'iwaliro.

Ng'otoileku ensonga eyindhi, abaana abaalina obukosefu bw'ensigo obw'amangu, ebitundhu 95% baali mu katyabaga kanene okugemebwa obulwaire bw'ensigo obutaghona oba okufa. Abaana abaghona okufa obukosefu bw'ensigo, ebitundhu 95% baali mu katyabaga kanene ak'okugemebwa obulwaire bw'ensigo obutaghona.

Okuvunhula. Mu kibiindha eky'abaana bano abaanhonherezebwaku, kyaazuulibwa nti obukosefu bw'ensigo obw'amangu butera inho okuvaamu okufa oluvainhuma nga wabise eibbanga, ekiraga nti obukosefu bw'ensigo obubi einho kabonero akatalaga kubaagho kwa buzibu bwonka, aye n'akabi ak'okufa, era ekyongera okulaga obunafu obuli mu kwidhandaba endwaire embi einho mu malwaliro agali mu bifo omwaganibwa einho ensiri edhileeta omusudha.

Ebigambo ebikulu. Okukosebwa kw'ensigo okwamaanhi; omusudha gw'ensiri ogw'amaanhi; okubagho kw'abaana okutuuka ku myaka etaanu; obulwaire bwensigo obutaghona; ebiva mukukola ekintu eby'ekiseera ekighanvu; enfuna entonho.

Okuvhuunula kunho mu lulimi olusoga kwaghebwaayo abaghandiisi era niiffe tukukoppolola nga bwe kwatughebwa. Ttikwekenhenhiezeibwa bakugu. Emitendera edhigobererwa kampuni eyetibwa Lancet mu kwekebeedha, okulongoosa n'okuteekateeka ebighandiikiibwa dhigobeleilwa mu kkopi eyasooka yonka eri mu lulimi olungereza; era eyo nerina okwidhulizibwa mu kiwandiiko kino.
