# Supplementary material for: Long-term mortality and risk of chronic kidney disease in children following severe malaria complicated by acute kidney injury: a prospective observational study
Source: Lancet Glob Health. Author manuscript; Available in PMC 2026 Nov 2. (PMC13726406; doi:10.1016/j.langlo.2026.103984)
Supplement: Supplementary file 4 [file NIHMS2215924-supplement-4.pdf]

THE LANCET
Global Health

## Supplementary appendix 4

This translation in Langi was submitted by the authors and we reproduce it as supplied. Ithas not been peer reviewed. *The Lancet*' editorial processes have only been applied to theoriginal in English, which should serve as reference for this manuscript.

Coc ogonyo i leb lango ococ adongo en ocwalo eka wan ote cwalo oko iyore kit ame omio kede. Jo angeo leb lango adongo dongo pwod pe ru ongio. Wopo yore ducu tye iye Lancet ingiyo, medo rwomere kede yubu gin ocoo piny ame man pwod tye ka i leb munu; ento tye acalo anyut me jami ocoo.

Supplement to: Mellencamp KA, Namazzi R, Batte A, et al. Long-term mortality and risk of chronic kidney disease in children following severe malaria complicated by acute kidney injury: a prospective observational study. *Lancet Glob Health* 2026. https://doi.org/10.1016/j.langlo.2026.103984

## Langi Summary


### Kop acego

**Acaki.** Wane angalura atek bedo acalo adwogi me two atipa adwong adwonga ikom atin, ento adwogi mere iyi anyim nyangere ilobe acente gi tye piny kede idyere dyere. Wan nwongo tye omito niang ikom ngo atimere ikare me anyim iyi akina wane angalura otino, kite ame angalura tio kede medo kede too ame bedo atye inge two atipa abedo adwong ikom dano.

**Kit ame otimo kede.** Otio kede ngec oya ibot gurup aryo me otino atye iyi Uganda (cako idwete 6 tunu kede iyi 12) ame ogamo gi idakatal pi two atipa me odio gi atek iyi akina mwaka 2008 kede 2017. Otino ame ame okwanyo remo gi ote gwoko ame nwongo tye kede jami opimo akwako mot cam obin omedo pi ngiyo cura me jami apol atye iye. Otino ame obin obedo kwo obin oketo gi iyi ikweda me lubu yor gi cako imwaka 2020 tunu iyi mwaka 2023, ame ote bino pimo kite ame angalura gi tye atic kede kun oneno dang wel jo ame tye kwo kun otio kede yore me nyango kwon jami apol tuno naka ka jami mogo otimere me mwaka acalo kare me geco ka dano romo bedo kede two angalura nyo too oko. Cura ame oyungo tye iye jami ocoo ikare me gamo jo ame tye iye mwaka, nga ibedo, bori alubere kede mwaka, kan ikweda tye atimere iye, dul ene itye iye, dul me twoo atipa aporere two atipa me kango otino, two atipa atwoo remo, two atipa ayelo yoo yamo, kangogo ame kelo wano atek kede cilim.

**Adwogi me ikweda.** Iyi acaki cura me poko nuco rwom mere imalo kede nuco rwomere ping me mwaka 2·5 (1·8–3·5) 57·8% (622/1077) obedo coo, kede 42·2% (455/1077) obedo anyira, kede ret iyi anglura te bedo 40% (431/1077) me otino ducu. Akato poko nuco rwom mere imalo kede nuco rwomere ping) me mwaka 6·5 (3·7–8·8), 13·6% me otino obin otoo oko, kun aromo nucu too oko inge kwanyo gi idakatal. Pimm mede mere kede adwogi onwongo tye malo iyi akina otino atye kede ret me angalura (3·14, 95% 2·23–4·43), ame kero adwong me too en okelo (3·36, 95% 2·22–5·10) ame omede ibedo malo inge ret me angalura (4·53, 95% 1·80–11·37). Otino ame ocang ikom ret me angalura onwongo tye kede kero adit me bedo kede ret ame rii iyi angalura (1·77, 95% 1·07–2·93), ento onwongo pire pe tek ingo yiko (1·47, 95% 0·84–2·56).

**Gonyo adwogi me ikweda**. Iyi gurup me otino ni, awano me angalura onwongo tye okubere kede too ikare me anyim, ame cimo ni awano me angalura acako weko dano bedo agoro te too oko ame dang yutu kite ame yore apapat me cango two atipa goro kede kan ame two man tye adwong iye.

**Nyig kop apire tek.** Wane angalura; two atipa; kwo atin; two angalura ame rii; adwogi ame rii; cente anunok.


Coc ogonyo i leb lango ococ adongo en ocwalo eka wan ote cwalo oko iyore kit ame omio kede. Jo angeo leb lango adongo dongo pwod pe ru ongio. Wopo yore ducu tye iye Lancet ingiyo, medo rwomere kede yubu gin ocoo piny ame man pwod tye ka i leb munu; ento tye acalo anyut me jami ocoo.
