# Supplementary material for: Long-term mortality and risk of chronic kidney disease in children following severe malaria complicated by acute kidney injury: a prospective observational study
Source: Lancet Glob Health. Author manuscript; Available in PMC 2026 Nov 2. (PMC13726406; doi:10.1016/j.langlo.2026.103984)
Supplement: Supplementary file 5 [file NIHMS2215924-supplement-5.pdf]

THE LANCET
Global Health

Supplementary appendix 5

This appendix formed part of the original submission and has been peer reviewed.
We post it as supplied by the authors.

Supplement to: Mellencamp KA, Namazzi R, Batte A, et al. Long-term mortality and risk of chronic kidney disease in children following severe malaria complicated by acute kidney injury: a prospective observational study. *Lancet Glob Health* 2026. https://doi.org/10.1016/j.langlo.2026.103984

Supplementary Appendix

Table of Contents

Supplementary methods 1
Kidney replacement therapy (KRT) in Uganda 1
Measurement of kidney function 1
Assessment of mortality 2
Statistical analysis 2
Supplementary analysis 4
Supplementary Table S1. Inclusion and exclusion criteria and clinical management for severe malaria according to study cohort 4
Figure S1. Flowchart of study enrolment and follow-up according to prior cohort 6
Supplementary Table S2. Baseline demographic, clinical and laboratory characteristics of children with severe malaria according to whether children were lost to follow-up/withdrew 7
Supplementary Table S3. Median estimated glomerular filtration rates over follow-up according to subsequent mortality 8
Supplementary Table S4. Long-term major adverse kidney events (MAKE-LT), chronic kidney disease (CKD), mortality, or all-cause hospitalizations over follow-up according to study cohort, study site, and participant sex 9
Supplementary Table S5. CKD risk categories (KDIGO) at the follow-up study using cystatin C-based eGFR equation and creatinine-cystatin C combined eGFR equation, with associated kidney dysfunction according to AKI on admission for severe malaria 10
Figure S2. Survival probabilities according to AKI on admission for severe malaria across years since enrolment and age 11
Supplementary Table S6. Comparison of focal covariate effects on eGFR trajectories from two modeling approaches 12
Supplementary Table S7. Cox proportional hazards regression models evaluating mortality risk over follow-up based on AKI on admission for severe malaria using different imputed exit ages for children lost to follow-up between studies 13
Supplementary Table S8. Baseline demographic, clinical and laboratory characteristics among children with AKI on admission for severe malaria according to death over follow-up 14
Supplementary references 15

Supplementary methods

Kidney replacement therapy (KRT) in Uganda

Haemodialysis was initiated at Mulago National Referral Hospital (MNRH) in 2012. Between 2016 and 2019, dialysis services were moved to Kiruddu Referral Hospital in Kampala, requiring patients to be transferred to another facility for hemodialysis, which was provided as a subsidized fee-for-service on adult wards. There was one paediatric nephrologist in Uganda until 2014 until they left the country. There was a gap with no paediatric nephrologist available for two years. In 2016 and then 2020, additional pediatric nephrologists were trained and started working at MNRH. In 2019, dialysis services were transferred back to MNRH. For Cohort 1 (2008–2013, dialysis was unavailable, and study testing identified AKI after the initial hospitalization, because in-hospital creatinine monitoring was not routine. For Cohort 2 (2014–2017), point-of-care measurements of creatinine, electrolytes, and blood gases were obtained, and pediatric nephrologists were consulted when severe AKI was identified, and medical management was initiated. While a proportion of children had laboratory findings on admission (uremia, severe acidosis, hyperkalemia) that may have required dialysis, no participants received dialysis during the study enrollment period due to limited capacity to transfer critically ill children, most deaths occurring within 24 hours, and limitations in dialysis access. For participants enrolled at the Jinja site, dialysis access was even more limited as it required transfer to Kampala for services (100km). While dialysis centers were expanded to include other districts in 2020. We are unaware of any participants requiring dialysis or transplant over follow-up. Transplant services are available in Uganda in a limited capacity, with the first adult transplant occurring in 2023 and the first pediatric transplant in 2025 for a sixteen-year-old with kidney failure following severe malaria-associated AKI.

Measurement of kidney function

Creatinine was tested on cryopreserved plasma samples using the Beckman Coulter AU680 with a modified Jaffe method in Cohort 1 at ARUP laboratories (Utah, USA) and cryopreserved serum samples using the Beckman Coulter AU5822 in Cohort 2 (Beckman Coulter, Brea, California) at Indiana University Pathology Laboratory (Indianapolis, USA). Creatinine was tested at enrollment, 12- and 24-month follow-up visits in Cohort 1 and enrollment, 24-hour, 1- and 12-month follow-up visits in Cohort 2. In Cohort 2, creatinine testing at 12 months was restricted to a subsample of children enrolled in a sub-study using an enzymatic method (iSTAT, Abbot). Therefore, we applied a linear calibration equation to adjust the enzymatic values, incorporating both fixed and proportional bias adjustments to produce Jaffe-equivalent estimates at 12 months among children from Cohort 2.[1,2]

In the follow-up study, serum and urine creatinine were tested on cryopreserved samples in batches using a Cobas 6000 (c501 module, Roche Diagnostics) at the MNRH clinical chemistry laboratory using the modified Jaffe method, which is traceable to an IDMS standard. Urine albumin was tested using an immunoturbidimetric assay on a Cobas 6000. Serum cystatin C was measured using a Quantikine ELISA (R&D Systems, Minneapolis, MN) at 1:30 dilution with a reported range of 0·06 to 16 mg/L with pooled serum controls and commercial controls on every plate. The assay has been correlated against the Cystatin C reference standard provided by the Joint Research Centre Institute for Reference Material and Measurements (Catalog # ERM-DA471/ IFCC) with a slope of 1·07 and a R^2 value of 0·998.

Assessment of mortality

Within each prior study, deaths were confirmed if they occurred during the initial hospitalization or during a hospital readmission over study follow-up. Deaths occurring outside of the hospital were confirmed with the child's caregiver. Mortality that occurred between the end of the prior studies and the follow-up study was captured when home visitors contacted the participants' caregivers about participation in the follow-up study. Study personnel attempted to contact caregivers via home visits or phone calls using numbers on file from the prior studies. If the caregiver was successfully contacted and stated the participant was deceased, consent was sought to complete a verbal autopsy to record the cause and timing of the death. If the caregiver could not be contacted or, upon contact, could not provide the vital status of the participant, the participant was considered lost to follow up. Attrition from the study due to loss to follow-up or withdrawal was similar between the cohorts (25.3% in Cohort 1 vs. 20.6% in Cohort 2, p=0·07), the majority of which (72%) occurred between the prior studies and follow-up study. Overall, differences in the ability to track participants from the two prior studies were not significant, though slightly higher in Cohort 1 which included children from Kampala only during an earlier period (2008-2013). The longer follow-up period and potentially more mobile study population exclusively from peri-urban Kampala could have contributed to the small but insignificant differences in tracking participants across prior studies.

Statistical analysis

Cox proportional hazards models and Kaplan-Meier curves with age as timescale

Children entered the risk set at their age at enrolment and exited at their age at death, age at enrolment in the follow-up study, or age at loss to follow-up or withdrawal. Age was rounded to the nearest year before truncation, with results robust to rounding to the nearest half-year. Exact exit ages were unknown for children lost to follow-up after completing their prior study. In these children, we estimated the exit age by taking their age at prior study completion and adding half the average time between prior study

completion and follow-up study enrollment. This approximation represented the child's expected age at the midpoint of the follow-up period for their prior cohort. As a sensitivity analysis of our handling of unknown exit ages, we also censored children lost to follow-up at (a) prior study completion or (b) the start of the follow-up study, using children's known ages at prior study completion or children's expected ages at follow-up study enrollment, respectively (Table S8).

Restricted Mean Survival Time (RMST)

To compare survival ages between children with and without AKI, we calculated the restricted mean survival time up to age 10. The restricted mean survival time summarizes the average survival time within a specified age window and is particularly useful for long-term follow-up with censored data. Age 10 was chosen as the cutoff because few deaths occurred beyond this point, and sufficient numbers remained at risk to ensure reliable group comparisons.

Linear Mixed-Effects Regression

Descriptive analysis revealed that serial eGFR values were consistently lower among children who died within one year of enrollment compared to those who survived (Table S3). To account for potential attrition bias, confounders, and within-subject variability over time, an adjusted linear mixed-effects (LME) regression with a random intercept and slope was used to assess changes in mean eGFR by AKI status over time using serial measurements at scheduled follow-up visits and lagged between-visit dropout indicators.[3] As a sensitivity analysis of our handling of attrition, we also fit a joint longitudinal-survival model using R v4·4·3 (R Foundation),[4] linking the LME model for eGFR to a Cox proportional hazards model for time to death or loss to follow-up. To compare the two methods, we adapted the original model using lagged dropout indicators by fitting time continuously (Table S6).

Supplementary analysis

Supplementary Table S1. Inclusion and exclusion criteria and clinical management for severe malaria according to study cohort

	Cohort 1 (2008–2013)	Cohort 2 (2014–2017)
**Inclusion criteria**	**Cerebral malaria (CM):** Coma (Blantyre coma score ≤2) with the presence of *Plasmodium falciparum* by blood smear and no other known cause of the coma (e.g., meningitis, a prolonged postictal state or hypoglycaemia-associated coma reversed by glucose infusion).<br><br>**Severe malarial anaemia (SMA):** Presence of *P. falciparum* by blood smear with a haemoglobin level ≤5 g/dL.<br><br>***Note:*** Children could meet multiple criteria and were enrolled in groups based on severity of criteria met: CM > SMA. For example, a child in coma with severe anaemia was enrolled in the CM group.	**All children with SM:** *P. falciparum* by rapid diagnostic test or blood smear and one or more of the following complications: coma (Blantyre coma score ≤2), respiratory distress (deep acidotic breathing or lower chest wall retractions), multiple seizures (≥2 generalized seizures in 24 hours or a seizure >30 minutes in duration), severe anaemia (haemoglobin <5 g/dL) or prostration (≥1 year, unable to sit unsupported or stand; <1 year, unable to breastfeed).<br><br>***Note:*** Children could meet multiple criteria and were enrolled in groups based on severity of criteria met: CM > respiratory distress > complicated seizures > SMA > prostration. For example, a child with respiratory distress and severe anaemia was enrolled in the respiratory distress group.
**Exclusion criteria**	**All children with severe malaria (SM):** Known chronic illness requiring medical care, known developmental delay or history of coma, head trauma, hospitalisation for malnutrition or cerebral palsy.<br><br>**Additional for SMA:** Impaired consciousness at physical examination, other	**All children with SM:** Known developmental delay, known chronic illness requiring medical care or history of cerebral palsy, coma, head trauma, or hospitalisation for malnutrition.

	clinical evidence of central nervous system disease or >1 seizure before admission.	
**Clinical management for severe malaria**	Managed according to the Ugandan Ministry of Health treatment guidelines current at the time of the study, including intravenous quinine treatment followed by oral quinine for severe malaria during hospital admission and artemisinin combination therapy for outpatient follow-up therapy. Toward the end of the study, the treatment shifted to the use of parenteral artemisinin-based therapies for the treatment of severe malaria following the 2011 World Health Organization recommendation of injectable artesunate as the first-line treatment for severe malaria. All children with a haemoglobin level <5 g/dL received a blood transfusion.	Managed according to the Ugandan Ministry of Health treatment guidelines current at the time of the study, including intravenous artesunate followed by an oral course of artemisinin-combination therapy. All children with a haemoglobin level <5 g/dL or <6 g/dL with signs of respiratory distress received a blood transfusion.

Figure S1. Flowchart of study enrolment and follow-up according to prior cohort

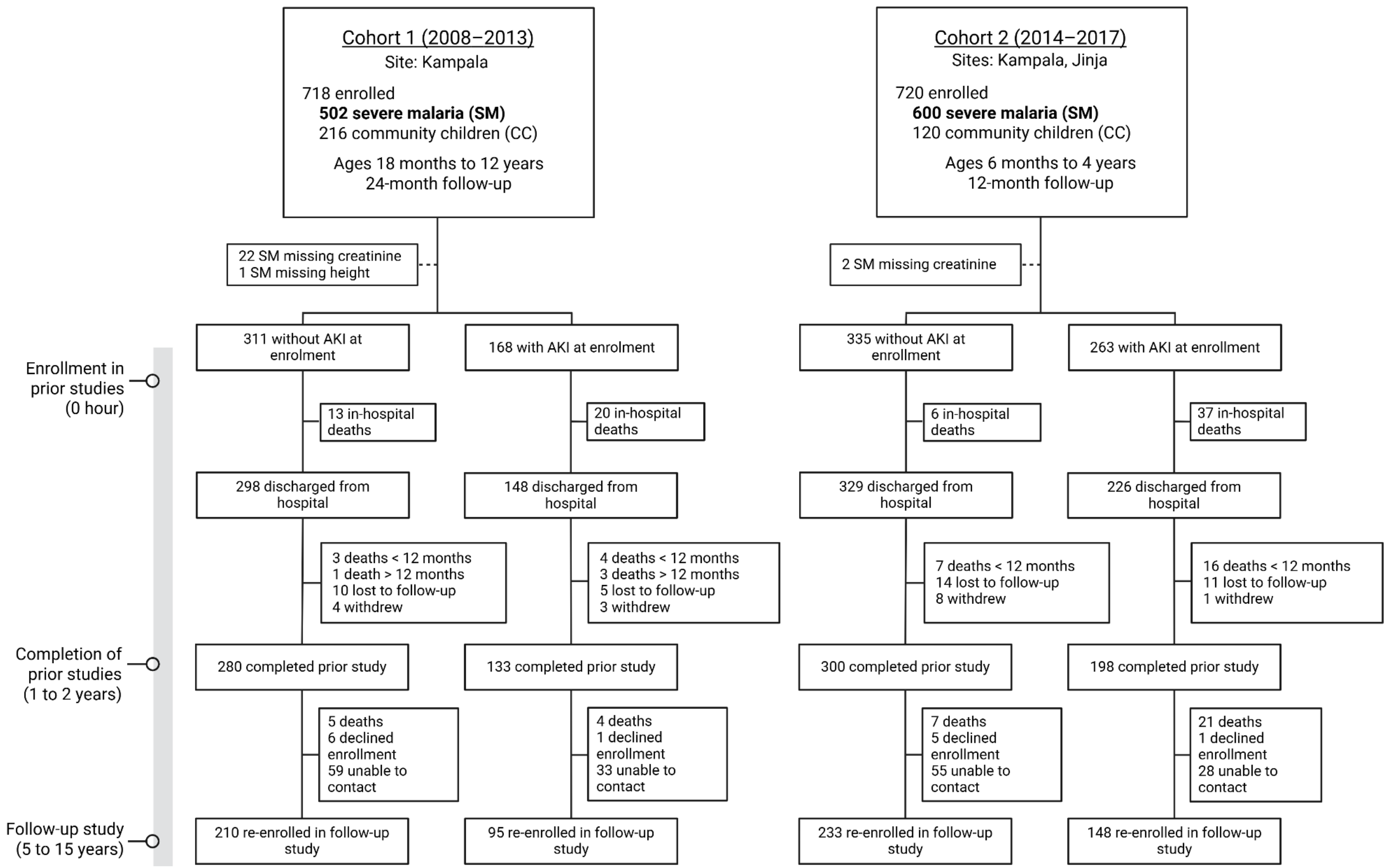

Supplementary Table S2. Baseline demographic, clinical and laboratory characteristics of children with severe malaria according to whether children were lost to follow-up/withdrew

	N	Lost to follow-up/withdrew	N	Not lost to follow-up	P value
**Demographics**					
Age, years	244	2·6 (1·8–3·4)	833	2·5 (1·7–3·5)	0·38
Sex	244		833		0·55
Male, n (%)		145 (59·4)		477 (57·3)	
Female, n (%)		99 (40·6)		356 (42·7)	
Site, Kampala, n (%)	244	37 (15·6)	833	231 (27·7)	<0·001
Weight-for-age z-score	244	-1·1 (-1·9 to -0·25)	822	-1·1 (-1·8 to -0·40)	0·93
Height-for-age z-score	244	-1·2 (-2·1 to -0·35)	833	-1·1 (-1·9 to -0·30)	0·42
**Clinical characteristics**					
Duration of fever, days	244	3 (2–5)	822	3 (2–4)	0·20
Axillary temperature, °C	244	37·8 (37·0–38·8)	833	37·6 (36·9–38·5)	0·049
Coma, n (%)	244	77 (31·6)	833	268 (32·2)	0·86
Multiple seizures, n (%)	243	51 (21·0)	830	176 (21·2)	0·94
Respiratory distress, n (%)	244	28 (11·5)	833	182 (21·9)	<0·001
Acute kidney injury, n (%)	244	83 (34·0)	833	348 (41·8)	0·03
Blackwater fever, n (%)	244	50 (20·5)	830	174 (21·0)	0·87
Severe anaemia, n (%)	244	109 (44·7)	830	411 (49·5)	0·18
Prostration, n (%)	244	174 (71·3)	833	616 (74·0)	0.41
Jaundice, n (%)	244	81 (33·2)	832	343 (41·2)	0·02
HIV infection, n (%)	239	8 (3·4)	810	16 (2·0)	0·21
**Laboratory findings**					
White blood cells, x10^3/µL	241	11·1 (8·0–15·9)	825	11·2 (8·2–18·0)	0·15
Platelet count, x10^3/µL	241	94 (51–170)	826	108 (56–203)	0·08
Haemoglobin, g/dL	244	5·5 (3·9–8·3)	830	5·1 (3·7–7·7)	0·07
Parasite density, parasites/µL	241	41021 (3296–223175)	826	28105 (1510–166473)	0·06
Plasma HRP-2, ng/mL	240	2311 (631–4886)	831	2069 (470–5153)	0·35
Bacteremia, n (%)	227	9 (4·0)	784	41 (5·2)	0·49
Lactate, mmol/L	230	3·8 (2·6–6·6)	787	4·4 (2·4–7·8)	0·06
BUN, mg/dL	243	13 (8–19)	829	14 (9–22)	0·01
Creatinine, mg/dL	244	0·35 (0·29–0·46)	833	0·38 (0·29–0·50)	0·02

Data presented as median (IQR) unless indicated otherwise. P values obtained from Wilcoxon rank-sum tests for continuous variables and Pearson's Chi-squared tests for categorical variables.

Supplementary Table S3. Median estimated glomerular filtration rates over follow-up according to subsequent mortality

	N	Died after visit	N	Survived to final visit or censored	P value	Cohort
Enrollment	147	62·9 (41·0–77·0)	930	86·2 (66·3–108·9)	<0·001	1, 2
24 hours	57	83·4 (35·2–116·4)	395	116·0 (89·5–139·6)	<0·001	2
1-month follow-up	41	102·1 (72·4–129·6)	421	115·4 (92·5–136·6)	0·02	2
1-year follow-up	17	119·7 (108·2–128·5)	488	110·5 (96·5–124·7)	0·19	1, 2
2-year follow-up	7	108·1 (104·5–147·8)	356	110·2 (97·2–128·8)	0·40	1

Data presented as median (IQR). P values obtained from Wilcoxon rank-sum tests. Glomerular filtration rates estimated using the “CKiD under 25” (U25) equation.

Supplementary Table S4. Long-term major adverse kidney events (MAKE-LT), chronic kidney disease (CKD), mortality, or all-cause hospitalizations over follow-up according to study cohort, study site, and participant sex

Outcome	(a) Cohort		(a) Site		(c) Sex	
	**Cohort 2 vs. Cohort 1**	**P value**	**Jinja vs. Kampala**	**P value**	**Female vs. Male**	**P value**
MAKE-LT (aOR)	1·51 (0·89–2·54)	0·13	3·64 (2·32–5·72)	<0·001	1·83 (1·29–2·58)	0·001
CKD (aOR)	0·71 (0·26–1·97)	0·52	5·76 (2·42–13·73)	<0·001	3·47 (1·99–6·04)	<0·001
Mortality (aHR)	1·96 (1.15–3.34)	0·01	1·73 (1·12–2·67)	0·01	1·19 (0·84–1·69)	0·34
All-cause hospitalization (aIRR)	2·73 (2·07–3·59)	<0·001	3·24 (2·73–3·85)	<0·001	0·97 (0·85–1·12)	0·71

Estimates reflect the adjusted odds ratios (aOR), hazard ratios (aHR), or incidence rate ratios (aIRR) and 95% CI for (a) study cohort, (b) study site, or (c) participant sex. aOR were obtained from a single logistic regression of kidney outcomes among eligible participants (MAKE-LT, all participants; CKD, participants with at least two eGFR measurements ≥90 days apart), aHR were obtained from a single Cox proportional hazards regression of mortality from enrolment with age as the timescale (all participants), and aIRR were obtained from a single Poisson regression of all-cause hospitalizations over prior study follow-up with a person-time offset (discharged participants), all adjusted for the focal variables (cohort, site, and sex) as well as baseline AKI, age, height-for-age z-score, severe malaria group, and HIV status.

Supplementary Table S5. CKD risk categories (KDIGO) at the follow-up study using cystatin C-based eGFR equation and creatinine-cystatin C combined eGFR equation, with associated kidney dysfunction according to AKI on admission for severe malaria

**Cystatin C-based eGFR categories (mL/min/1·73m²)**	**Albuminuria categories (uACR, mg/mmol)** Normal to mildly increased (<3)	Moderately increased (3-29)	Severely increased (≥30)	**Kidney dysfunction n/N (%) AKI vs. No AKI**
Normal or high (≥90)	249	71	3	45/211 (21·3) vs. 88/401 (22·0), p=0·86
Mildly decreased (60-89)	230	42	1	
Mildly to moderately decreased (45-59)	11	1	0	
Moderately to severely decreased (30-44)	4	0	0	
Severely decreased (15-29)	0	0	0	
Kidney failure (<15)	0	0	0	
**Serum creatinine-cystatin C combined eGFR categories (mL/min/1·73m²)**	**Albuminuria categories (uACR, mg/mmol)** Normal to mildly increased (<3)	Moderately increased (3-29)	Severely increased (≥30)	**Kidney dysfunction AKI vs. No AKI**
Normal or high (≥90)	360	88	5	44/226 (19·5) vs. 88/460 (20·5), p=0·76
Mildly decreased (60-89)	164	35	0	
Mildly to moderately decreased (45-59)	4	0	0	
Moderately to severely decreased (30-44)	0	0	0	
Severely decreased (15-29)	0	0	0	
Kidney failure (<15)	0	0	0	

Number of children in each chronic kidney disease (CKD) risk category based on Kidney Disease: Improving Global Outcomes (KDIGO) consensus criteria, determined by the combination of estimated glomerular filtration rates (eGFR), using cystatin C only and creatinine-cystatin C combined equations, and albuminuria, using urine albumin to creatinine ratios (uACR). Persistent kidney dysfunction among survivors enrolled in the follow-up study was defined as a moderately increased or greater risk of CKD (i.e., eGFR <60 mL/min/1·73m² or uACR ≥3 mg/mmol).

Figure S2. Survival probabilities according to AKI on admission for severe malaria across years since enrolment and age

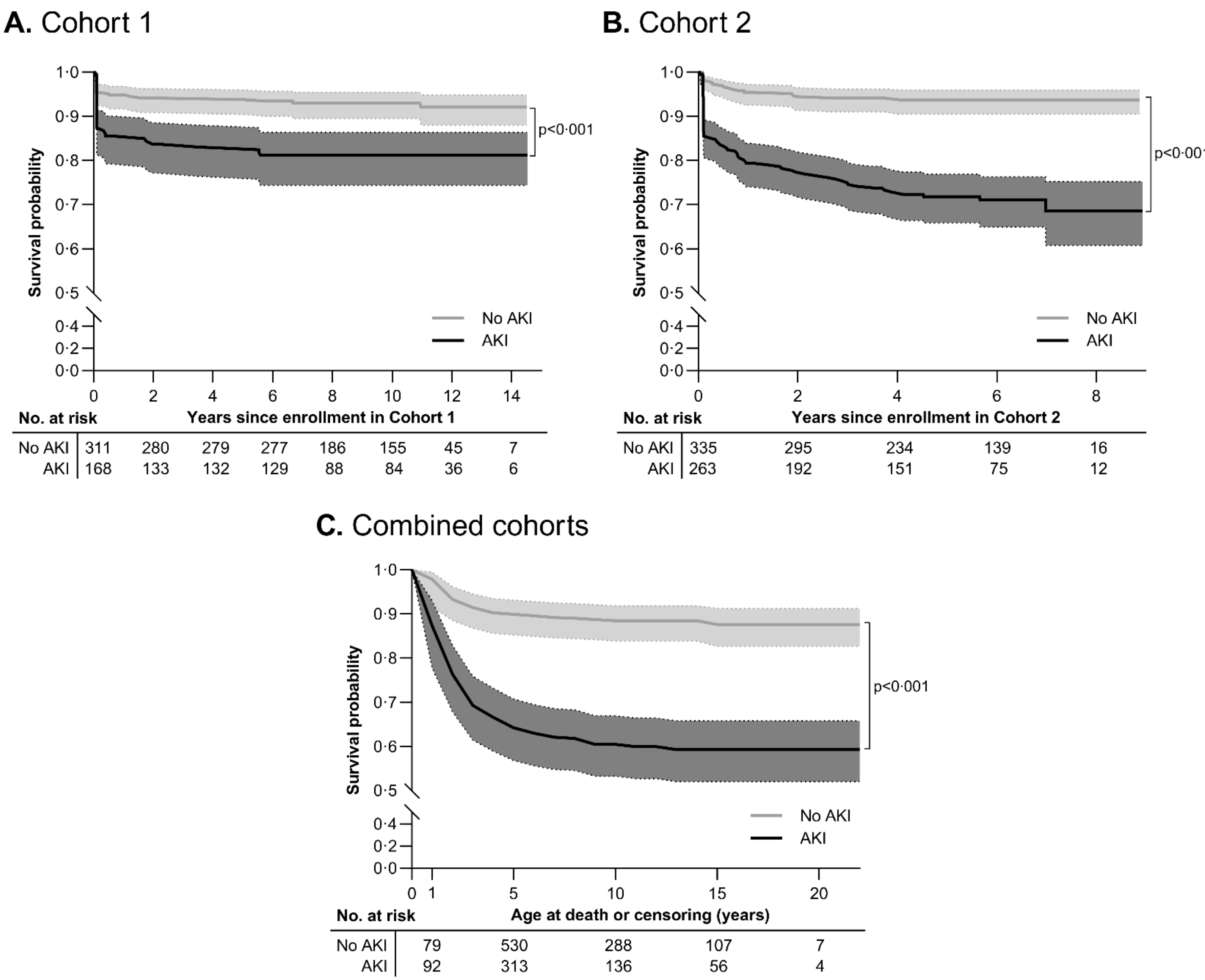

Supplementary Table S6. Comparison of focal covariate effects on eGFR trajectories from two modeling approaches

	Primary LME regression		Joint longitudinal-survival model			
	With dropout indicators		LME regression submodel		Cox regression submodel	
**Focal covariate**	**aβ (95% CI)**	**P value**	**aβ (95% CI)**	**P value**	**aHR (95% CI)**	**P value**
AKI	-56·59 (-59·85 to -53·34)	<0·001	-54·85 (-58·56 to -51·15)	<0·001	1·50 (1·32–1·71)	<0·001
Visit time	0·75 (0·22–1·27)	0·01	-0·10 (-0·69–0·49)	0·73	--	--
AKI × Visit time	10·51 (9·64–11·37)	<0·001	10·05 (9·08–11·01)	<0·001	--	--
α from joint model	--	--	0·004, p=0·24			

Estimates are presented as adjusted beta coefficients (aβ 95% CI) with p values, representing the overall effect of each focal covariate on eGFR across the full follow-up period. Left panel presents estimates from the primary linear mixed-effects (LME) regression, which included lagged, between-visit indicators for death and loss to follow-up. The middle panel shows the LME submodel from the joint longitudinal-survival analysis (JM package, R), which treated follow-up time as continuous and linked longitudinal eGFR to a Cox proportional hazards regression model (right panel) for time to death or dropout via shared random effects. All models used continuous visit time for comparison and were adjusted for age, sex, height-for-age z-score, study site, study cohort, severe malaria group, and HIV status. The LME models additionally accounted for a subject-specific intercept and random slope. The association parameter (α) from the joint model quantifies the extent to which longitudinal changes in eGFR predict the hazard of dropout or death. Abbreviations: aHR, adjusted hazard ratio; AKI, acute kidney injury.

Supplementary Table S7. Cox proportional hazards regression models evaluating mortality risk over follow-up based on AKI on admission for severe malaria using different imputed exit ages for children lost to follow-up between studies

Cox PH Model	AKI HR (95% CI)	P value
Imputed exit age = age at prior study completion		
Among all enrolled children (N=1077)		
Unadjusted	3·82 (2·67–5·47)	<0·001
Adjusted	3·30 (2·18–5·00)	<0·001
Among in-hospital survivors (N=1001)		
Unadjusted	3·46 (2·10–5·69)	<0·001
Adjusted	3·09 (1·82–5·25)	<0·001
Among 1-year survivors (N=920)		
Unadjusted	3·72 (1·92–7·18)	<0·001
Adjusted	3·57 (1·78–7·18)	<0·001
Among 2-year survivors (N=811)		
Unadjusted	4·78 (2·01–11·39)	<0·001
Adjusted	4·68 (1·85–11·86)	0·001
Imputed exit age = expected age at follow-up study enrollment		
Among all enrolled children (N=1077)		
Unadjusted	3·86 (2·70–5·52)	<0·001
Adjusted	3·35 (2·21–5·08)	<0·001
Among in-hospital survivors (N=1001)		
Unadjusted	3·50 (2·13–5·76)	<0·001
Adjusted	3·07 (1·81–5·21)	<0·001
Among 1-year survivors (N=920)		
Unadjusted	4·10 (2·08–8·06)	<0·001
Adjusted	3·74 (1·83–7·64)	<0·001
Among 2-year survivors (N=900)		
Unadjusted	4·89 (2·05–11·63)	<0·001
Adjusted	4·44 (1·78–11·09)	0·001

Hazard ratios (HR) and 95% CIs from three sets of Cox proportional hazards (PH) regression models of death according to imputation of exit ages for children lost to follow-up between prior study completion and follow-up study enrollment. Hazard ratios are presented both unadjusted and adjusted for enrollment characteristics, including sex, height-for-age z-score, study site, study cohort, severe malaria manifestation, and HIV status.

Supplementary Table S8. Baseline demographic, clinical and laboratory characteristics among children with AKI on admission for severe malaria according to death over follow-up

	N	Died	N	Survived	P value
**Demographics**					
Age, years	105	2·2 (1·7–3·3)	326	2·4 (1·7–3·4)	0·47
Sex	105		326		0·40
Male, n (%)		56 (53·3)		189 (58·0)	
Female, n (%)		49 (46·7)		137 (42·0)	
Site, Kampala, n (%)	105	56 (53·3)	326	223 (68·4)	0·005
Weight-for-age z-score	99	-1·2 (-2·0 to -0·48)	325	-1·1 (-1·8 to -0·38)	0·36
Height-for-age z-score	105	-1·3 (-1·8 to -0·84)	326	-0·99 (-2·0 to -0·29)	0·14
**Clinical characteristics**					
Duration of fever, days	102	3 (2–4)	323	3 (2–4)	0·09
Axillary temperature, °C	105	37·6 (36·7–38·3)	326	37·7 (37·0–38·6)	0·10
Coma, n (%)	105	53 (50·5)	326	113 (34·7)	0·004
Multiple seizures, n (%)	104	32 (30·8)	325	82 (25·2)	0·27
Respiratory distress, n (%)	105	58 (55·2)	326	82 (25·2)	<0·001
Blackwater fever, n (%)	105	45 (42·9)	325	82 (25·2)	0·001
Severe anaemia, n (%)	103	60 (58·3)	326	145 (44·5)	0·02
Prostration, n (%)	105	97 (92·4)	326	269 (82·5)	0·01
Jaundice, n (%)	104	52 (50·0)	326	136 (41·7)	0·14
HIV infection, n (%)	94	3 (3·2)	323	6 (1·9)	0·43
**Laboratory findings**					
White blood cells, $x10^3$/µL	102	16·1 (9·9–30·2)	322	12·1 (8·6–18·8)	0·002
Platelet count, $x10^3$/µL	102	128 (46–212)	322	90 (47–184)	0·17
Haemoglobin, g/dL	103	402 (3·2–7·2)	326	5·5 (3·9–8·2)	0·001
Parasite density, parasites/µL	103	11135 (47–136265)	321	34188 (1963–249278)	0·01
Plasma HRP-2, ng/mL	105	2194 (214–5522)	325	3367 (963–6972)	0·01
Bacteremia, n (%)	101	6 (5·9)	310	16 (5·2)	0·76
Lactate, mmol/L	100	8·1 (3·9–13·5)	312	4·7 (2·8–8·3)	<0·001
BUN, mg/dL	104	28·5 (19·6–45·5)	326	20·9 (14–31)	<0·001
Creatinine, mg/dL	105	0·60 (0·47–0·93)	326	0·52 (0·44–0·64)	<0·001

Data presented as median (IQR) unless indicated otherwise. P values obtained from Wilcoxon rank-sum tests for continuous variables and Pearson's Chi-squared or Fisher's exact tests for categorical variables, where appropriate. Presented for descriptive purposes; not included in main analysis.

Supplementary references

1. Batte A, Murphy KJ, Namazzi R, et al. Evaluating kidney function using a point-of-care creatinine test in Ugandan children with severe malaria: a prospective cohort study. *BMC Nephrol* 2021; **22**(1): 369.
2. Fabian J, Kalyesubula R, Mkandawire J, et al. Measurement of kidney function in Malawi, South Africa, and Uganda: a multicentre cohort study. *The Lancet Global Health* 2022; **10**(8): e1159-e69.
3. Muthén B, Asparouhov T, Hunter AM, Leuchter AF. Growth modeling with nonignorable dropout: alternative analyses of the STAR* D antidepressant trial. *Psychological methods* 2011; **16**(1): 17.
4. Rizopoulos D. Joint models for longitudinal and time-to-event data: With applications in R: CRC press; 2012.
