# Supplementary material for: Long-term mortality and risk of chronic kidney disease in children following severe malaria complicated by acute kidney injury: a prospective observational study
Source: Lancet Glob Health. Author manuscript; Available in PMC 2026 Nov 2. (PMC13726406; doi:10.1016/j.langlo.2026.103984)
Supplement: Supplementary file 6 [file NIHMS2215924-supplement-6.pdf]

THE LANCET
Global Health

Supplementary appendix 6

This appendix formed part of the original submission and has been peer reviewed. We post it as supplied by the authors.

Supplement to: Mellencamp KA, Namazzi R, Batte A, et al. Long-term mortality and risk of chronic kidney disease in children following severe malaria complicated by acute kidney injury: a prospective observational study. *Lancet Glob Health* 2026. https://doi.org/10.1016/j.langlo.2026.103984

Equitable Partnership Declaration

If any questions do not apply to your study, please indicate "N/A" for "not applicable.
For more information on how to complete this form see the Information for Authors document.

Researcher considerations

1. Please detail the involvement that researchers who are based in the country or countries of study had during a) study design; b) clinical study processes, such as processing blood samples, prescribing medication, or patient recruitment; c) data interpretation; and d) manuscript preparation, commenting on all aspects. If they were not involved in any of these aspects, please explain why.

This should include a thorough description of their leadership roles in the study. Are local researchers named in the author list or the acknowledgements, or are they not mentioned at all (and, if not, why)? Please also describe the involvement of early career researchers based in the location of the study. Some of this information might be repeated from the Contributors section in the manuscript. Note: we adhere to ICMJE authorship criteria for naming authors on a paper.

**a) Study design:**<br>Dr. Robert Opoka was involved in study design, participant recruitment, and clinical care, ensuring oversight of patient care across both cohorts and the follow-up study. Dr. Ruth Namazzi coordinated cohort 2, supported participant recruitment and clinical care during follow-up, and was involved in the clinical care and referral of participants from the follow-up study. Dr. Paul Bangirana is a clinical psychologist who was involved in participant recruitment and follow-up for both cohorts and was the site principal investigator for the follow-up study. Dr. Anthony Batte is a paediatric nephrologist who was involved in care of participants with severe AKI in cohort 2 after returning from his training in 2016 and was involved in the design of the renal follow-up studies. He coordinated the referral and clinical care of participants found to have CKD throughout follow-up and ensured they were registered with the hospital renal clinics for ongoing clinical care.
**b) Clinical study processes:**<br>Clinical study processes were led by Drs. Robert Opoka and Ruth Namazzi who oversaw participant recruitment, clinical care, and research procedures for the studies. Caroline Kazinga was involved in clinical specimen processing for the follow-up study, and coordinated the urine albumin and creatinine testing, and conducted the Cystatin C testing.
**c) Data interpretation:**<br>Drs. Opoka, Namazzi, Batte and Kalyesubula were involved in reviewing all data and results and supported in the interpretation of the data and guided on additional testing and analyses.
**d) Manuscript preparation:**<br>All Ugandan authors contributed to manuscript preparation, critically reviewed, and approved the final manuscript.

2. How was funding used to remunerate and enhance the skills of researchers in the countries of study? And how was funding used to improve research infrastructure at the study sites?

   Potentially effective investments into long-term skills and opportunities within local institutions could include training or mentorship in analytical techniques and manuscript writing, opportunities to lead all or specific aspects of the study, financial remuneration rather than requiring volunteers, and other professional development and educational opportunities.

   Improvements to research infrastructure could include funding extended trial designs (eg, platform trials), establishment of long-term contracts for research staff, building research facilities, and setting up local control of funding allocation.

**Skills:**<br><br>All research staff were financially remunerated through study funding, ensuring that participation did not rely on unpaid or volunteer labour. Often, this resulted in long-term contracts for research staff and opportunities for additional training and skill development. In parallel, funding supported formal training programmes, including NIH Fogarty International Center (D43) training grants and fellowships, which enabled Ugandan trainees to pursue MSc, PhD, and postdoctoral training. These programmes provided mentorship and hands-on experience in data management, biostatistics, molecular biology and immunology, and clinical research methods.<br><br>In addition to formal degree training, local investigators and trainees were actively engaged in all aspects of the research process, including study design, implementation, data analysis, and manuscript preparation. Several Ugandan investigators who contributed to this work advanced their academic and clinical careers through these opportunities, including obtaining advanced clinical qualifications and research degrees (e.g., PhD and MMed), supporting progression to independent research leadership.
**Research infrastructure:**<br><br>Study funding also contributed to the development of long-term research infrastructure through the establishment and support of Global Health Uganda (GHU), a locally based not-for-profit research organisation co-led by Ugandan investigators. GHU provides administrative, regulatory, and operational support for research studies conducted in Uganda and enables local oversight of research activities.<br><br>Investment in infrastructure included strengthening laboratory capacity, establishing data management systems and data centres, and supporting clinical research platforms at participating sites. Laboratory analyses for this study were conducted through the GHU–Indiana University CHILD laboratory in Uganda, which provides in-country biomarker testing capacity and serves as a training platform for MSc and PhD students.<br><br>Together, these investments supported both immediate study implementation and longer-term capacity for locally led research, contributing to sustained research independence and infrastructure development within Uganda.

3. How did you safeguard the researchers who implemented the study?

Please describe how you guaranteed safe working conditions for study staff, including provision of appropriate personal protective equipment, protection from violence, and prevention of overworking.

All study staff were employed through Global Health Uganda, a locally based research organisation that operates under Ugandan labour laws and maintains a dedicated human resources structure to oversee staff welfare, contracts, and working conditions. Staff were embedded within established hospital systems at participating sites, ensuring alignment with local clinical governance and institutional oversight.<br><br>Safe working conditions were prioritised through multiple mechanisms. All staff received fair and locally appropriate compensation, formal contracts, and access to organisational support through the HR team. Study activities were conducted in collaboration with clinical sites that adhered to national safety standards, including infection prevention and control protocols.<br><br>Personal protective equipment (PPE) was provided to all staff in accordance with the level of clinical exposure, with enhanced PPE and additional safety measures implemented during periods of COVID-19 transmission in line with national and institutional guidelines. Staff were trained in infection prevention, safe clinical practices, and study-specific procedures.<br><br>To protect staff wellbeing, workloads were monitored by local study leadership to prevent excessive working hours, and responsibilities were distributed across teams to avoid overburdening individuals. Regular team meetings and supervision structures were in place to identify and address any concerns related to safety, workload, or working conditions. Staff also had access to institutional channels for reporting concerns confidentially.<br><br>All study procedures were conducted in accordance with local regulatory requirements and ethical approvals, and no incidents of violence or unsafe working conditions were reported during the study period.

Benefits to the communities and regions of study

4. How does the study address the research and policy priorities of its location?

How were the local priorities determined and then used to inform the research question? Who decided which priorities to take forward? Which elements of the study address those priorities?

The studies underlying this work were designed and implemented in partnership with Ugandan investigators based at Makerere University and affiliated clinical sites, who provided leadership in identifying locally relevant research priorities. These priorities were informed by direct clinical experience managing children with severe malaria, as well as ongoing engagement with patients, caregivers, trainees, and healthcare providers within participating hospitals, and policy makers at the national level.<br><br>Local priorities were further refined through the conduct of the parent cohort studies, which incorporated multiple research approaches, including longitudinal follow-up and mixed-methods components that captured patient and community experiences. These efforts

highlighted key gaps in care, including high post-discharge morbidity and mortality, and limited understanding of long-term outcomes following severe malaria and its complications.

Each successive cohort study was designed as an extension of prior work, allowing locally identified priorities to iteratively shape the research agenda. In particular, emerging evidence from earlier studies conducted by the team identified acute kidney injury (AKI) as a common complication of severe malaria and a potential contributor to adverse outcomes.

This manuscript builds directly on those locally generated priorities by incorporating extended follow-up of existing cohorts to evaluate long-term survival and kidney health. The focus on long-term outcomes reflects concerns raised by local investigators and clinicians regarding the lack of post-discharge surveillance and the potential for unrecognised long-term complications in children recovering from severe malaria.

Decisions regarding study priorities, design, and implementation were made collaboratively, with substantial leadership from local investigators at all stages. The resulting work addresses locally relevant clinical and policy questions, including the need for improved recognition of high-risk children and the integration of follow-up care into existing health systems.

5. How will research products be shared in the community of study?

For instance, will you be providing written or oral layperson summaries for non-academic information sharing? Will study data be made available to institutions in the region(s) of study? The Lancet Group *encourages authors to translate the summary (abstract) into relevant languages after paper editing; do you intend to translate your summary?*

Findings from this study will be shared with both academic and community stakeholders in Uganda through multiple approaches tailored to different audiences. Results will be disseminated to participating clinical sites through presentations, reports, and ongoing collaborative meetings, ensuring that findings can inform clinical practice and future research locally.

For community engagement, we will provide accessible summaries of study findings in lay language for participants, caregivers, and local communities, delivered through established community engagement structures and study follow-up networks. These may include verbal dissemination through community meetings and written summaries where appropriate.

We intend to translate key summaries of the study findings into locally relevant languages from the clinical sites following publication, in line with Lancet Group recommendations, to support broader accessibility beyond English-speaking audiences.

Study data and findings will remain accessible to collaborating institutions in Uganda, with local investigators maintaining active involvement in data interpretation, secondary analyses, and future research initiatives. Data sharing will be conducted in accordance with ethical approvals and data governance frameworks, with a focus on supporting locally led research.

In addition, findings will be shared with policymakers and stakeholders involved in child health and malaria programmes to support integration of study insights into local health systems, particularly with respect to post-discharge care and long-term follow-up.

6. How were individuals, communities, and environments protected from harm?

a) *How did you ensure that sensitive patient data were handled safely and respectfully? Was there any potential for stigma or discrimination against participants arising from any of the procedures or outcomes of the study?*

All study procedures were conducted in accordance with Good Clinical Practice (GCP) guidelines and were approved by relevant institutional review boards in Uganda and collaborating institutions. These frameworks ensured that participant rights, safety, and confidentiality were prioritised throughout the study.

Sensitive patient data were handled using secure and confidential processes. Physical records were stored in locked facilities with controlled access, and electronic data were maintained in password-protected databases with access restricted to authorised study personnel only. Data handling procedures complied with local regulatory requirements and institutional data governance policies, ensuring that participant information was managed safely and respectfully.

Participants received clinical care in accordance with national treatment guidelines, with study teams working closely with local hospital staff to ensure appropriate management of illness. Follow-up visits were conducted in designated study spaces to maintain privacy and confidentiality.

Potential risks of stigma or discrimination were minimised through integration of study activities within routine clinical care and through respectful engagement with participants and caregivers. There were no study procedures that singled out participants in a way that would increase the risk of stigma. For the follow-up study, visits occurred at the research offices rather than a hospital setting. High retention and follow-up rates, including participant return for unscheduled sick visits, indicate strong trust in the study teams and acceptance within the community.

Close collaboration with local hospitals and sustained engagement with clinical staff helped ensure that participants were treated equitably and without discrimination. No instances of breaches of confidentiality were reported during the study period.

b) *Might any of the tests be experienced as invasive or culturally insensitive?*

Study procedures included blood draws, medical history, and physical examinations, all of which are standard components of clinical care for children with severe malaria in these settings. While blood sampling may be considered minimally invasive, it was performed by trained clinical staff using established protocols to ensure safety, minimise discomfort, and maintain respect for participants.

All procedures were explained to caregivers, and written informed consent was obtained prior to participation, and assent was obtained in participants aged 8 and older in accordance with local guidelines. Study activities were conducted within familiar clinical environments and aligned with local standards of care, which supported acceptability.

No procedures were identified as culturally insensitive, and there were no reports of participant refusal or withdrawal related to the nature of the study procedures. High retention and continued engagement with follow-up visits further indicate that the procedures were well accepted by participants and their caregivers.

c) *How did you determine that work was sensitive to traditions, restrictions, and considerations of all cultural and religious groups in the study population?*

Cultural and religious sensitivity was ensured through close collaboration with local investigators, clinicians, and study staff who are embedded within the communities served by the participating hospitals. The study team included a diverse local workforce familiar with the languages, cultural practices, and social norms of the study population, which informed all aspects of participant engagement and study implementation.

Participants were recruited through public referral hospitals that serve diverse populations reflective of the local community, ensuring that study procedures were conducted within established and culturally appropriate clinical environments. Study processes, including consent and follow-up, were conducted in local languages and adapted as needed to ensure understanding and respect for participants and their caregivers.

Ongoing engagement with clinical staff and participants during the study allowed the team to identify and address any concerns related to cultural or religious considerations. No concerns related to cultural insensitivity were raised during the study, and the strong participant retention and follow-up suggest that the study procedures were acceptable across the communities involved.

d) *Were biowaste and radioactive waste disposed of in accordance with local laws?*

Only biological (biohazardous) waste was generated as part of this study; no radioactive materials were used. All biohazardous waste was handled and disposed of in accordance with local regulations and institutional policies at participating clinical and laboratory sites.

Study staff were trained in safe handling, segregation, and disposal of biological materials, and all procedures followed established standard operating procedures (SOPs). Waste generated during clinical and laboratory activities was managed within existing hospital and laboratory systems, including appropriate containment, transport, and disposal processes.

Licensed biohazard waste management services were engaged where required to ensure safe and compliant disposal. These practices ensured that all biological waste was handled responsibly and posed no risk to staff, participants, or the surrounding environment.

e) *Were any structures built that would have impacted members of the community or the environment (such as handwashing facilities in a public space)? If so, how did you ensure that you had appropriate community buy-in?*

Limited infrastructure development was undertaken at one participating clinical site to support study activities. These improvements were implemented within existing hospital facilities and were designed to strengthen clinical and research capacity without disrupting surrounding communities or the environment.

All infrastructure developments were carried out following consultation with and approval from hospital leadership and in accordance with local regulatory and institutional guidelines. Decisions regarding these improvements were led by local investigators and site leadership to ensure alignment with institutional priorities and community needs.

Because these developments were confined to hospital settings and integrated into existing systems, they did not impact public spaces or require broader community modification. As such, they were well accepted by local stakeholders and contributed to strengthening the clinical and research environment at the site.

f) *How might the study have impacted existing health-care resources (such as staff workloads, use of equipment that is typically employed elsewhere, or reallocation of public funds)?*

The study was designed to minimise strain on existing health-care resources while contributing to local capacity strengthening. Study activities were integrated within participating hospitals in a way that avoided overburdening routine clinical services.

To prevent increased workload on existing staff, dedicated study personnel were hired and embedded within hospital teams to support patient enrolment, follow-up, and data collection. These staff also contributed to broader clinical and operational activities, helping to offset workload demands rather than shifting them onto existing personnel.

Where specialised equipment or diagnostics were required, these were procured through study funding and placed within participating facilities to expand local capacity rather than divert resources from routine care. In settings where certain tests were not routinely available, analyses were either supported through study-funded infrastructure or outsourced to avoid placing additional demand on local laboratory systems. Formal agreements (e.g., memoranda of understanding) were established with participating sites to ensure appropriate use and sustainability of resources.

7. Confirm that local ethics review was sought, and please provide the approval number. If not sought, please explain why.

Local ethical approval was obtained from Makerere University School of Medicine Research Ethics Committee (SOMREC) for cohort 1 (REC REF No. 2008-033), cohort 2 (REC REF No. 2013-141), and the follow-up study (REC REF No. 2018-091). Administrative clearance was obtained from each participating clinical site. Local regulatory approval was obtained from the Uganda National Council of Science and Technology: cohort 1 (HS 432), cohort 2 (HS 1522), follow-up study (HS 2468).

Secondary analyses

8. Have the data analysed in your study been extracted from another source, such as a national survey, rather than being directly collected by the authors of this paper?

No

If the authors of this paper were not involved in data collection, how were the findings interpreted with sufficient contextual knowledge?
The Lancet Group *believe contextual understanding is crucial for informed data analysis and interpretation*.

All authors were involved across multiple stages of the research process, including study design, implementation, data analysis, and interpretation, ensuring continuity and deep contextual understanding of the study population and setting.

Local investigators based in Uganda played central roles throughout the study, contributing clinical expertise grounded in direct experience managing children with severe malaria and its complications. This ensured that data interpretation was informed by local clinical practice, health-system context, and patient realities.

As the research programme evolved, the team was intentionally expanded to include additional expertise to strengthen interpretation of findings. This included incorporating adult nephrology expertise to support a life-course perspective on kidney injury and its long-term consequences, allowing findings to be interpreted beyond a solely paediatric framework.

Together, this multidisciplinary and locally anchored approach ensured that analyses and conclusions were grounded in both contextual knowledge and relevant clinical expertise, supporting appropriate and meaningful interpretation of the results.

---

9. Please provide the title (eg, Dr/Prof, Mr/Mrs/Ms/Mx), name, and email address of an author who can be contacted about this statement.

Name: Chandy John
Email: chjohn@iu.edu

10. Finally, please provide the title and name of an author from one country of study who has seen and approved this form.

Name: Dr. Ruth Namazzi
